# Psychological drivers and barriers affecting students' sustained usage intention of AIGC tools in educational settings: an integrated perspective of Uses and Gratifications Theory and Perceived Value Theory

**DOI:** 10.3389/fpsyg.2026.1892349

**Published:** 2026-08-21

**Authors:** Qianghong Huang, Junping Xu, Ru Zhang, Ge Song

**Affiliations:** 1 College of Art and Design, Nanning University, Nanning, China; 2 Academy of Fine Arts, Beihai University of Art and Design, Beihai, China; 3 College of Media and International Culture, Zhejiang University, Hangzhou, China

**Keywords:** AIGC tools, educational settings, perceived trust, Perceived Value Theory, sustainable digital learning, sustained usage intention, Uses and Gratifications Theory

## Abstract

**Introduction:**

This study systematically examines the drivers and barriers influencing students' sustained usage intention toward artificial intelligence-generated content (AIGC) tools in educational settings. As AIGC tools are increasingly introduced into teaching and learning activities, understanding how students accept, evaluate, and continue to use these tools is essential for promoting sustainable digital learning in higher education. Grounded in the Uses and Gratifications Theory and the Perceived Value Theory, this study develops an integrated model to explain students' continuance intention toward AIGC tools from both motivational and inhibitory perspectives.

**Methods:**

Questionnaire data were collected from 528 Chinese university students and analyzed using partial least squares structural equation modeling (PLS-SEM). The study also examined the moderating role of perceived trust and conducted multi-group analysis to explore differences across student groups.

**Results:**

Content gratification, utilitarian gratification, and social gratification were significantly positively associated with continued usage intention. Among these factors, learning needs and perceived usefulness showed the strongest positive associations, whereas enjoyment within hedonic gratification showed no significant association. Perceived risk and perceived cost were significantly negatively associated with continued usage intention. Perceived trust significantly moderated the relationships between key gratifications, perceived sacrifices, and sustained usage intention. Multi-group analysis further revealed that postgraduates placed greater emphasis on information retrieval, undergraduates relied more on trust to reduce uncertainty, and infrequent users were more strongly associated with novelty.

**Discussion:**

These findings contribute to understanding the psychological mechanisms underlying students' sustained use of AIGC tools and provide practical implications for their responsible integration into higher education. Universities and educators should enhance the functional and learning-support value of AIGC tools while establishing clear usage guidelines, strengthening privacy protection, reducing cost barriers, and providing differentiated instructional support.

## 1 Introduction

As artificial intelligence continues to be widely applied, artificial intelligence generated content (AIGC) has achieved remarkable results as a disruptive technology in improving labor efficiency, reducing costs and promoting employment innovation (Zhang and Lu, 2021). It is predicted that by 2030, 3–14% of the global workforce will face the need to switch careers or upgrade their skills due to the development of artificial intelligence (Manyika, 2017). Therefore, there is a growing need for graduates possessing sustainable development competencies in all walks of life (Desha et al., 2009; Wang and Wang, 2022). Especially within the field of education, AI's integration can help students in improving digital competence, critical reasoning, and problem-solving abilities, thus boosting their employability (Gulati et al., 2024).

This rapid penetration of AIGC technology has made a significant impact on the art world, covering design, painting, music, poetry, film, and many other aspects (Hong and Curran, 2019; Liu, 2020; Cetinic and She, 2022; Cao et al., 2023). Studies indicate that the use of AIGC not only exerts a profound influence on the traditional labor market, but also creates new opportunities and challenges for related industries (Goralski and Tan, 2020; Peres et al., 2023). In art and design fields, students' proficiency with AIGC technology has become crucial for improving their market competitiveness (Kerr-Phillips and Thomas, 2009). If higher education institutions fall behind in creating AIGC programs, students may find it difficult to gain the necessary skills, which will subsequently impact their employability and creativity (Halpern, 2014; Nagel and Pidaparti, 2016). This will not only reduce students' competitiveness in the workplace, but may also affect the opportunities for collaboration and the reputation of the educational institution with industry (Shaikh and Khoja, 2011; Cooper, 2023).

Despite the many benefits that AIGC tools bring to higher education, they also pose risks and challenges, including the threat to scholarly ethics, concerns over plagiarism, as well as bias in evaluation (Farrelly and Baker, 2023). If students abuse or rely indiscriminately on these tools, their capacity for critical reasoning and problem-solving may be undermined (Jo A., 2023). Therefore, besides focusing on their potential benefits, it is essential to address the moral concerns and potential dangers posed by generative AI (Alasadi and Baiz, 2023). Students' usage behavior not only affects individual learning efficiency and employability, but also provides valuable feedback for the sustainable integration of AIGC tools into teaching and learning environments (Wang and Chen, 2024). Therefore, research on AIGC use in higher education should not only examine its functional benefits, but also consider the psychological, ethical, and practical barriers that may affect students' sustained usage intention (Baytak, 2023; Prasad Agrawal, 2024).

The use and gratification theory (UGT) is an effective framework for studying students continued use of AIGC tools (Katz et al., 1973b). The theory has found extensive application in studies of computer-mediated communication, emerging platforms, and learning on mobile devices (Shukla, 2021; Chang et al., 2022). In contrast to other technology acceptance models like TAM, UTAUT, and TPB, the advantage of UGT is that it not only focuses on users' acceptance of technology, but also pays special attention to the emotional factors and satisfaction, which are key parts of users' personal and social experiences and are often neglected in traditional technology diffusion theory (Gao, 2023). Therefore, UGT provides an effective framework for studying users' sustained usage intention.

However, the current literature has some shortcomings in using UGT to study AIGC tools. Many studies focus on general satisfaction or technology acceptance, but pay less attention to the specific types of gratification that students may obtain from AIGC tools in educational contexts. Although models such as TAM and UTAUT have provided important explanations of technology acceptance, their emphasis is mainly placed on functional usefulness, ease of use, social influence, and facilitating conditions. In contrast, students' sustained use of AIGC tools may involve a broader psychological evaluation, including learning-related needs, enjoyment, achievement, intelligent interaction, employability expectations, and concerns about risk and cost. Therefore, this study uses UGT as the main framework for explaining students' positive gratifications from AIGC tool use.

In addition, existing research on ChatGPT or related AI tools has often emphasized users' acceptance and perceived usefulness, while the possible negative evaluations associated with continued use have received relatively less attention. To address this issue, this study incorporates perceived value theory into the analysis by introducing perceived sacrifice, including perceived risk and perceived cost (Arslan, 2020). When considering whether to continue using AIGC tools, students not only evaluate the gratifications they obtain, but also consider potential risks and costs. Furthermore, this study introduces perceived trust as a moderating factor to examine how trust shapes the relationships between content gratification, perceived sacrifice, and sustained usage intention. Therefore, this study develops an integrated model based on UGT and PVT to explain students' sustained usage intention toward AIGC tools in educational settings. The findings provide empirical evidence for universities and educators seeking to integrate AIGC tools into teaching and learning in a responsible, sustainable, and student-centered manner.

## 2 Literature review

### 2.1 The integration of AIGC tools in educational settings

With the rapid development of AIGC technology, its application in higher education has gradually expanded to multiple fields such as text generation, image creation, audio and animation production, design assistance, and information search. In this study, AIGC tools refer to generative artificial intelligence applications that can assist with the production, transformation, or refinement of text, images, design ideas, learning materials, and other digital content based on user prompts or instructions. Rather than focusing on one specific platform, this study examines students' general experience of using AIGC tools for learning-related purposes in higher education settings. These applications not only enrich the presentation of educational content, but also greatly enhance the creativity of digital media creation (Chen et al., 2024). In tertiary education, university students, as primary beneficiaries, have a significant need for information gathering, problem-solving, academic writing, creative practice, and innovative tasks, which are highly aligned with the multi-modal creation features of AIGC tools. This makes them a major user group (Ren et al., 2023).

One of the core strengths of AIGC tools in education is their strong productivity support capabilities. By simplifying repetitive tasks, these tools not only improve the personalisation and adaptability within the learning process, but likewise enable students to better cope with complex learning challenges and promote the growth of advanced cognitive skills (Qadir, 2023). These tools effectively promote students' innovative thinking by providing personalized learning resources, supporting the solution of academic problems, generating programming languages, and inspiring design (Farrokhnia et al., 2024). In this study, “educational settings” specifically refers to higher education learning contexts in which students use AIGC tools to support coursework, information retrieval, academic writing, creative ideation, design-related learning tasks, project development, and skill improvement.

Nevertheless, the usage of AIGC tools within education brings certain possible hazards, especially in terms of academic self-discipline, innovation capabilities, and data privacy. Academics have noted that overreliance on these tools may lead to students losing the ability to think independently and inhibit the development of critical thinking, as well as ethical and privacy protection issues (Celik, 2023; Tlili et al., 2023). Therefore, although AIGC has revolutionized education, its use must carefully balance the benefits of technology with its potential negative effects (Alasadi and Baiz, 2023).

These benefits and risks suggest that the educational value of AIGC tools depends not only on their technical capabilities, but also on how they are integrated into teaching and learning practices. Therefore, identifying the factors that encourage or hinder students' continued use of AIGC tools is important for designing appropriate instructional strategies, usage guidelines, and support mechanisms in higher education.

### 2.2 Use and Gratification Theory

#### 2.2.1 The concept of Use and Gratification Theory

Use plus gratification theory (UGT) was first proposed by Katz et al. (1973a) to explain users' media consumption behavior from the viewpoint of socio-psychological needs. The theory posits that users are proactive and opt for particular technologies or devices according to their needs and desires, instead of being passive recipients of information. Users proactively engage with technology to fulfill personal motivations like gaining information, seeking enjoyment, and expressing identity (Katz et al., 1973a). UGT explains users' motives for using technology and how these motives are driven by and satisfied with needs (Valentine, 2013).

In today's digital era, the significance of UGT theory is growing more prominent. It not only emphasizes the practical attributes of technology, but also includes personal preferences, psychological needs, and social influences, which aids in understanding the motives and values behind users' behaviors, such as utilizing social platforms and emerging technologies (Geng et al., 2024). UGT has been widely used in mobile learning (Shukla, 2021), virtual reality (Cheng Y. et al., 2022), chat bots (Haberlin and Atkin, 2022), virtual assistants (Wald et al., 2023), and AI teachers (Niu et al., 2024), providing a powerful framework for studying user behavior with emerging technologies (Taherdoost, 2018).

The core concept of UGT is gratification, which is the process of users satisfying their needs through the use of media. When needs are met, users' sustained usage intention is enhanced. Therefore, the UGT theory is particularly applicable in the era of personalized Internet, helping to study user behavior in the post-acceptance stage and revealing whether they feel satisfied after accepting emerging technologies (Ruggiero, 2000). This study will assess students' motivation to use the AIGC tool based on UGT and explore whether their needs and expectations are met.

#### 2.2.2 Use and Gratification Theory types of gratification

Since the UGT was proposed, scholars have repeatedly explored the classification of gratifications (McQuail, 1972) initially categorized the gratifications of conventional media into four types: distraction (emotion release and escape), interpersonal connections (companionship and social utility), personal identity (self-reference and value enhancement), and monitoring (information acquisition). Subsequent studies further distinguished between seeking gratification, which refers to the gratification that users expect to obtain from future media use, and achieving gratification, which refers to the gratification that users actually obtain from past use (Palmgreen et al., 1980).

As the Internet has evolved, “content gratification” and “process gratification” have gradually been introduced. Content gratification denotes the fulfillment individuals obtain through using media to gather information and apply this in real-world situations, whereas process gratification arises from the enjoyment of casual exploration and site browsing (Hoffman and Novak, 1996). In addition, social gratification has also been added to the UGT category, reflecting the gratification and sense of achievement that users obtain through social interactions in the online environment (Stafford et al., 2004). Modern research has further expanded the types of gratification, such as coping needs, fantasy gratification, and social curiosity (Alzougool, 2018; Haberlin and Atkin, 2022). Although these new types make the UGT theory more adaptable, there is still some confusion in the existing classification framework. Table 1 shows the specific applications of UGT on different new media and technology platforms and the corresponding types of gratifications.

**Table 1:** The specific applications of UGT on different new media and technology platforms and the corresponding types of gratifications.

Number	Researcher	Platform	Use and gratification theory
1	Shukla, 2021	mobile learning	Cognitive needs gratification; Emotional needs gratification
2	Chang et al., 2022	Artificial intelligence learning application	Emotional needs gratification; Cognitive needs gratification; Social integration gratification; Personal integration gratification; Interaction gratification with artificial intelligence; Customization gratification
3	Gamage et al., 2022	WeChat	Content gratification: interactive content, user-generated content; Process gratification: instant access, convenience; Social gratification: self-expression, social influence
4	Gao, 2023	Intelligent mobile learning application	Content gratification: education; Hedonic gratification: sensory enjoyment; Utilitarian gratification: achievement; Technical gratification: intelligence, convenience; Social gratification: status
5	Masrom et al., 2024	Instagram	Social gratification; Content gratification; Entertainment Escape; Social presence
6	Baek and Kim, 2023	ChatGPT	Information search; Task efficiency; Personalization; Social interaction; Enjoyment
7	Xie et al., 2024	artificial intelligence chatbot	Hedonic gratification: entertainment; Utilitarian gratification: perceived usefulness; Technological gratification: moderate appeal, convenience;Social gratification: social interaction, social presence
8	Wu et al., 2024	Museum of the Metaverse	Hedonic gratification: entertainment, hope; Utilitarian gratification: information, self-presentation; Technological gratification: immersion, intelligent interaction; Social gratification: social interaction, social presence
9	Wei et al., 2024	Internet	Content gratification: information search, education, career opportunities; Process gratification: entertainment, escape, general flow, passage of time; Self-expression gratification: achievement/competitiveness, cognition/self-existence, self-expression, trends/novelty; Social gratification: communication, virtual socializing, socializing in general
10	Niu et al., 2024	Artificial intelligence educator	Information-seeking gratification; Social interaction gratification; Entertainment gratification
11	Le et al., 2024	ChatGPT	Demand for academic content creation; Demand for information search; Novelty

#### 2.2.3 Types of gratification in this study

Drawing upon previous studies, this study broadens the application of UGT by categorizing students' gratification from AIGC tool use into five key types: content gratification, hedonic gratification, utilitarian gratification, technological gratification, and social gratification. It should be noted that UGT and PVT constitute the main theoretical framework of this study. UGT is used to explain the positive gratifications students obtain from using AIGC tools, while PVT is introduced to capture the perceived sacrifices, such as risk and cost, that may inhibit sustained usage intention. The additional theories referenced in Table 2, including TAM, UTAUT, Flow Theory, Diffusion of Innovations, Achievement Goal Theory, and Social Cognitive Theory, are not treated as independent competing frameworks. Instead, they are used as conceptual references to support the selection and operationalization of specific constructs within the broader UGT-based gratification structure. This approach helps adapt UGT to the educational AIGC context, where students' sustained usage intention is shaped not only by general media gratifications, but also by learning usefulness, achievement, intelligent interaction, convenience, social influence, and employability-related expectations.

**Table 2:** Types of gratification and their factors.

Type of gratification	Specific structure	Conceptual reference	Definition
Content gratification	Information search	Uses and gratifications theory	Students actively seek and obtain relevant information through the AIGC tool to meet their learning needs.
	learning needs	Expectancy- value theory	Students' learning needs when using AIGC tools reflect their desire for knowledge and understanding.
Hedonic gratification	Enjoyment	Flow theory	The pleasure and gratification students experience when using the AIGC tool.
	Novelty	Diffusion of innovations	The uniqueness and freshness of the AIGC tool for students.
Utilitarian gratification	Perceived usefulness	TAM theory	Students' subjective evaluation of the help and value of the AIGC tool in the learning process.
	Sense of achievement	Achievement goal theory	The gratification and sense of self-realization students feel when they use the AIGC tool to complete learning tasks or goals.
Technological gratification	Intelligent interaction	User experience theory	The AIGC tool supports students' learning through intelligent interactive functions such as dialogue, real-time feedback, and personalized recommendations.
	Convenience	UTAUT theory	Students feel the convenience and efficiency when using the AIGC tool.
Social gratification	Social influence	UTAUT theory	When students use the AIGC tool, they are influenced by the views and attitudes of others in their social environment.
	Employability	Social cognitive theory	Students improve their employability and resilience within the job market through the use of AIGC tools.

Content gratification: Composed of information search and learning needs, it reflects the process of students obtaining information and satisfying their learning needs through the use of AIGC tools (Wei et al., 2024). Hedonic gratification: includes enjoyment and novelty, and describes the pleasurable experience and sense of novelty that students get when using AIGC tools (Le et al., 2024; Nguyen and Nguyen, 2024). Utilitarian gratification: consists of perceived usefulness and a sense of accomplishment, and assesses students' perception of the usefulness of AIGC tools in learning and their sense of self-realization after completing a task (Wei et al., 2024; Xie et al., 2024). Technical gratification: This includes intelligent interaction and convenience, highlighting the benefits of AIGC tools regarding interactivity and user-friendliness (Chang et al., 2022; Gao, 2023). Social gratification: This includes social influence and employability, focusing on the enhancement of social support and career competitiveness when students use AIGC tools (Gamage et al., 2022; Wei et al., 2024). Table 2 summarizes these gratification types and their constituent factors, detailing their theoretical basis and specific definitions.

### 2.3 Theory of perceived value

The theory of perceived value (PVT) suggests that the key to user acceptance of new technologies lies in their perceived value (Zhong and Chen, 2023). This value is primarily shaped by two components: perceived benefits and perceived sacrifices (Arslan, 2020). When accepting technology, users' perceived value is influenced by the combined impact of the advantages they gain, such as utility and pleasure, as well as the costs they bear, including cost and uncertainty (Kim et al., 2007). Research has demonstrated that perceived cost and perceived risk are critical factors in evaluating new technologies and significantly influence users' adoption decisions (Al-Saedi et al., 2020).

#### 2.3.1 Perceived risks

Perceived risks refer to users' expectations regarding the potential adverse effects of emerging technologies (Chao et al., 2016). This includes privacy issues, data security, system reliability, and potential over-reliance (Xiong et al., 2024). For instance, when using AIGC tools, students may become over-reliant due to the convenience of the tools. In particular, beginners may be inclined to directly copy the generated content without verification or thinking (Chen et al., 2021; Guo, 2023). This reliance may weaken students' cognitive abilities and initiative, which in turn raises questions regarding the standard of education (Ahmad et al., 2023).

The privacy risks of AI services have been widely discussed in many fields. For example, consumers are concerned about the misuse of personal data by chatbots (Sundar and Kim, 2019). With the development of AI mapping tools, users' concerns about privacy, security, copyright and originality are increasing (Chen et al., 2023). Studies suggest that as perceived risk increases, users' willingness to continue using these tools decreases (Song et al., 2022). This study defines perceived risk as the users' expectations when using AIGC tools, specifically addressing concerns such as privacy breaches, data security, system reliability, and over-reliance on technology.

#### 2.3.2 Perceived cost

Perceived cost pertains to the financial, temporal, and effort-related investment recognized by individuals during the use of new tools (Kim et al., 2007). In the context of the AIGC tool, perceived cost may include subscription fees, upgrade fees, and other expenses during use. For example, although ChatGPT-3.5 is free, a paid subscription is required for the more advanced ChatGPT-4 (George and George, 2023). Users evaluate these costs comprehensively when weighing the benefits and costs of technology. If the benefits outweigh the costs, users tend to persist with the technology; otherwise, if the costs prove too high, users may abandon or reconsider using it (Chunxiang, 2014; Hsu and Lin, 2015; Liao et al., 2022).

Studies have demonstrated that perceived cost represents an important element affecting user decisions, especially in educational environments where students often face limited budgets and need to balance paid tools with other necessary expenses (Wang et al., 2013; Chunxiang, 2014; Habibi and Rasoolimanesh, 2021). When the cost of AIGC tools increases and cannot be justified by providing unique value, users' attraction to them decreases.

### 2.4 Perceived trust

Perceived trust denotes users' trust regarding the reliability, efficiency, and data privacy protection associated with technology (Singh et al., 2021). Perceived trust serves as an essential element influencing individuals' acceptance of emerging technologies during the technology adoption process. It strengthens users' faith in the technology and reinforces their belief that the technology can effectively achieve their goals, thereby increasing their willingness to use it (Bauer, 1967; Lin et al., 2020).

As AI technology advances rapidly, users' trust toward these technologies is essential for their acceptance and sustained use (Kim et al., 2008). Research indicates that fostering perceived trust can help facilitate technology adoption. For instance, increasing confidence in AI-assisted programming tools can enhance users' belief in the tool's functions, thereby encouraging them to use it (Pan et al., 2024). Similar research has demonstrated that perceived trust strongly influences the willingness to keep using AI voice assistants and chatbots (Choung et al., 2023). Within this research, perceived trust functions as a moderating variable to explore its connection between content gratification, perceived sacrifice, and sustained usage intention.

## 3 Hypothesized development

This study integrates the Uses and Gratifications Theory with the Perceived Value Theory to develop a framework for exploring the determinants of students' continued use of AIGC tools in educational settings. In this context, continued use is not only a matter of individual technology adoption, but also reflects whether AIGC tools can be sustainably embedded into students' learning activities and institutional teaching practices.

**Figure 1:**
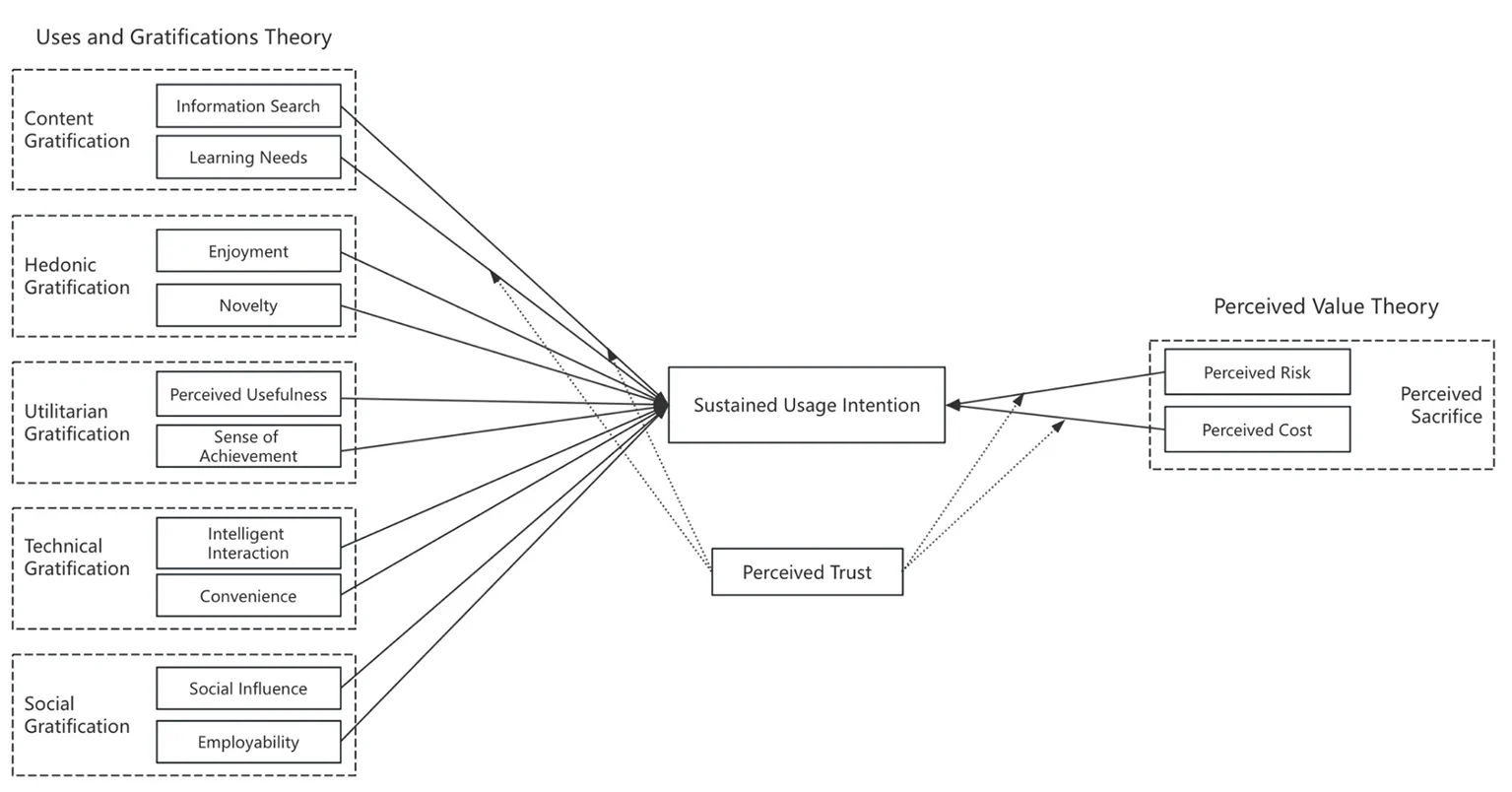
Conceptual model proposed.

The model consists of two types of influencing factors: positive influencing factors, which include content gratification, hedonic gratification, utilitarian gratification, technical gratification, and social gratification from the UGT. These factors emphasize the gratification students gain from using AIGC tools, which enhance their usage intention. The negative influencing factors are perceived risk and perceived cost from the PVT, which focus on issues students may face when using the tools, such as privacy breaches, technological dependence, and cost burdens. These issues may weaken their willingness to use the tools. Additionally, perceived trust acts as a moderating variable, regulating the relationship between content gratification, perceived sacrifice, and sustained usage intention. Sustained usage intention is the dependent variable, defined as students' future intention to continue using AIGC tools (Bhattacherjee, 2001). Based on these relationships, the proposed conceptual model is presented in Figure 1.

### 3.1 Use and Gratification Theory

#### 3.1.1 Content gratification

This research focuses primarily on content gratification as reflected in the two aspects of information search and learning needs. Information search refers to the active gathering of information from students during the learning process to meet their learning needs. Learning needs reflect students' desire for knowledge and the need to understand complex problems. Content satisfaction has been proven in empirical studies to enhance user engagement with online media and promote the effectiveness of online learning (Stafford et al., 2004; Li and Liu, 2023).

Utilizing AIGC tools like ChatGPT in education has significantly changed the way students acquire knowledge, providing learning support and improving learning efficiency (Rudolph et al., 2023). It not only simplifies the comprehension of intricate ideas and enhances students' overall learning process, but also cultivates their critical thinking through information feedback (Mollick and Mollick, 2022; Bell and Bell, 2023). In addition, ChatGPT not only helps students to get feedback on their assignments, but also saves instructors' time (ElSayary, 2024). Based on the above discussion, the current research proposes the following hypotheses:

**H1a:** Information search is positively associated with students' sustained usage intention of AIGC tools.
**H1b:** Learning needs is positively associated with students' sustained usage intention of AIGC tools.

#### 3.1.2 Hedonic gratification

For this research, hedonic gratification primarily manifests in enjoyment and novelty. Enjoyment refers to the pleasure and satisfaction that students experience during the learning process using the AIGC tool. Earlier research has indicated that hedonic motivation is a significant factor in users' sustained usage of mobile apps and digital media (Ryan and Deci, 2000). When users have a positive experience, their sustained usage intention is significantly enhanced (Gallego et al., 2016).

Novelty refers to the uniqueness and freshness that students experience when using AIGC tools. When technology is perceived as innovative or unique, user interest and enjoyment are stimulated, thereby enhancing satisfaction and increasing the intention for ongoing use (Antón et al., 2017). As shown in the study by Bui et al., students experienced novelty in their interactions with AI tutors, which increased their satisfaction and loyalty toward AI tutors (Bui et al., 2024). Related studies also show that novelty has a significant influence on technology adoption because new technologies bring more interesting and innovative experiences (Karjaluoto et al., 2019; Alzyoud et al., 2024). Building on the above analysis, this study presents the following hypotheses:

**H2a:** Enjoyment is positively associated with students' sustained usage intention of AIGC tools.
**H2b:** Novelty is positively associated with students' sustained usage intention of AIGC tools.

#### 3.1.3 Utilitarian gratification

Previous research has shown that utilitarian gratification is crucial in users' experience and engagement with emerging digital systems (Akdim et al., 2022). In this study, utilitarian gratification is mainly reflected in two aspects: perceived usefulness and sense of achievement. Perceived usefulness refers to students' subjective experience of the help and value that AIGC tools bring to the learning process. Numerous studies indicate that perceived usefulness represents a key determinant in users' continued engagement with technology (Choung et al., 2023). Perceived usefulness is regarded as crucial in the technology adoption process, especially in e-learning environments, where perceived usefulness greatly influences the willingness to continue using it (Saeed Al-Maroof et al., 2020).

Sense of achievement refers to the satisfaction and self-worth that students experience when using AIGC tools to complete learning tasks or achieve learning goals. Research indicates that sense of achievement serves as a strong predictor of users' intention to continue using (Wu et al., 2010; Gao, 2023). The role of sense of achievement in driving users' continued use has also been confirmed in studies such as smartphone applications (Mi et al., 2021). Building on the above analyses, this study puts forward the following hypotheses:

**H3a:** Perceived usefulness is positively associated with students' sustained usage intention of AIGC tools.
**H3b:** Sense of achievement is positively associated with students' sustained usage intention of AIGC tools.

#### 3.1.4 Technology gratification

Technology gratification has become a central topic of research in recent times, especially during the Industry 4.0 era, where innovative technologies provide ideal application ecosystems for various fields and promote the overall improvement of user experience. Research has demonstrated that technology gratification has a positive association with users' willingness to continue using it (Al-Shamaileh and Sutcliffe, 2023; Wu et al., 2024; Xie et al., 2024).

In this study, technology gratification is mainly reflected in two aspects: intelligent interaction and convenience. Intelligent interaction refers to the AIGC tool's ability to enhance students' learning experience through intelligent features (e.g., dialogue, real-time feedback, personalized suggestions) (Chen et al., 2024). Earlier research has suggested that the use of perceptual intelligence within personal intelligent agents significantly enhances user behavioral intentions and represents an essential element in the adoption of AI tools (Moussawi et al., 2023).

Convenience is defined as the ease and efficiency that students experience when using AIGC tools. Convenience is an important driver of Internet and mobile media use (Leung and Wei, 2000; Flanagin and Metzger, 2006). In learning management systems, convenience has been demonstrated to greatly enhance users' intention to continue using them (Bansah and Darko Agyei, 2022). Building on the above analyses, this study puts forward the following hypotheses:

**H4a:** Intelligent interaction is positively associated with students' sustained usage intention of AIGC tools.
**H4b:** Convenience is positively associated with students' sustained usage intention of AIGC tools.

#### 3.1.5 Social gratification

In this study, social gratification is mainly reflected in two aspects: social influence and employability. Social influence refers to the influence of students' perspectives and attitudes from others (e.g., classmates, teachers, family members, or the social environment) when using AIGC tools. Studies have shown that social influence has a significant impact on users' behavioral patterns in the early adoption stages of emerging technologies, where users tend to conform to group norms to gain group acceptance (Venkatesh et al., 2012; Adapa et al., 2018). In the context of generative artificial intelligence, social influence has a significant positive user behavioral intention effect (G. C et al., 2024).

Employability denotes students' capacity to enhance their competitiveness and adaptability in the labor market after using the AIGC tool (Overtoom, 2000). Research has shown that continuous learning and vocational competency development are essential to remain competitive in the employment landscape (De Vos et al., 2020). actively developing these competencies can improve job performance and promote personal career sustainability (Blokker et al., 2019). From the perspective of sustainable digital learning, employability also reflects students' expectations that AIGC tools can support long-term skill development, adaptive learning, and future career readiness.Based on the above theoretical background and research evidence, the following hypotheses are proposed in this study:

**H5a:** Social influences is positively associated with students' sustained usage intention of AIGC tools.
**H5b:** Employability is positively associated with students' sustained usage intention of AIGC tools.

### 3.2 Theory of perceived value

#### 3.2.1 Perceived risk

Perceived risk holds a significant role in the process of technology acceptance, being understood as the anticipations and concerns of users regarding the possible negative outcomes when thinking about adopting or using a particular technology (Kim et al., 2008). In this research, perceived risk is understood as the anticipations users have regarding the possible negative impacts when using the AIGC tool, including concerns about privacy disclosure, data security, system reliability, and potential overreliance. These perceived risks may reduce users' sustained usage intention of AIGC tools. Research shows that high perceived risks correlate with reduced technology adoption rates, while lower perceived risks correlate with greater technology acceptance (Dogra and Kaushal, 2021; Essel, 2022). During the initial stages of technology adoption, students may have doubts about the integrity and reliability of the technology, and these concerns could negatively impact their attitudes toward it. Building on the theoretical foundation and research evidence discussed above, this study proposes the following hypotheses:

**H6a:** Perceived risk is negatively associated with students' sustained usage intention of AIGC tools.

#### 3.2.2 Perceived cost

Perceived cost refers to the financial, time, and cognitive investments that users make when adopting and using new technologies (Xie et al., 2021). For AIGC tools, perceived cost includes subscription fees, upgrade fees, and any additional fees incurred during use. For example, basic versions such as ChatGPT-3.5 may be free, while ChatGPT4′s advanced features require a paid subscription (Frenkel and Emara, 2024). When evaluating a technology's perceived value, individuals consider if the advantages gained outweigh the associated costs; otherwise, they may reconsider or abandon the service (Alimamy and Gnoth, 2022). Earlier studies have shown a notable negative association between perceived cost and the willingness to embrace e-learning services (Twum et al., 2022). Furthermore, perceived cost serves as an important determinant of users' choices for online transactions and related activities (Jo H., 2023). Drawing on the theoretical and empirical findings above, we propose the following hypothesis:

**H6b:** Perceived cost is negatively associated with students' sustained usage intention of AIGC tools.

### 3.3 Perceived trust

Perceived trust is users' inclination to rely on and persist in using a technology based on their beliefs about its reliability efficiency, and data privacy protection (Rousseau et al., 1998). It holds a pivotal position within technology adoption and utilization, especially in the face of uncertainty and potential risks (Muir, 1987). Within the field of human-computer interaction, perceived trust typically signifies users' confidence in the AI system's suggestions and responses; for instance, anthropomorphic features (e.g., warmth and competence) of conversational AI have been shown to significantly enhance users' perceived trust (Cheng X. et al., 2022).

In e-commerce and online services, trust typically mitigates the effects of risk in two ways: one is to reduce the perception of risk: trust reduces the risk that users perceive in technology use, especially when faced with data breaches or privacy issues (Kelton et al., 2008). The other is to enhance behavioral intentions: trust perception directly promotes users' willingness to use and reduces the psychological burden caused by uncertainty and potential risks (Gefen and Straub, 2004). In this study, perceived trust is defined as users' trust in the reliability, efficiency, and data privacy protection of AIGC tools, which may reduce negative perceptions, such as perceived risk and perceived cost, and strengthen the positive role of content-related gratification.

However, perceived trust is not specified as a moderator for all paths in the model. Instead, it is theoretically positioned to moderate the relationships that are most closely related to information reliability, learning-related content gratification, and perceived sacrifice. Specifically, information search and learning needs depend heavily on whether students believe that AIGC tools can provide accurate, reliable, and useful learning support. When students have a higher level of trust in AIGC tools, the positive relationships between content-related gratification and sustained usage intention may become stronger. In addition, perceived risk and perceived cost represent the sacrifice side of students' evaluation. Higher perceived trust may reduce uncertainty and make students more willing to tolerate potential risks or costs when using AIGC tools. Therefore, this study examines the moderating role of perceived trust in the relationships between information search, learning needs, perceived risk, perceived cost, and sustained usage intention. Therefore, the following hypotheses are proposed in this study:

**H7a:** Perceived trust moderates between information search and sustained usage intentions
**H7b:** Perceived trust moderates between need to learn and sustained usage intentions
**H7c:** Perceived trust moderates the relationship between perceived risk and sustained usage intentions.
**H7d:** Perceived trust moderates the relationship between perceived cost and sustained usage intentions.

## 4 Methods

### 4.1 Questionnaire design

The questionnaire of this study was designed to comprehensively collect relevant data from the respondents and to effectively measure the study variables. The first section of the questionnaire gathered basic demographic information from the respondents, including gender, age, educational level, experience with the AIGC tool, and its usage frequency. To ensure the validity of the questionnaire, the second section focused on measuring 14 variables, each using scales adapted from prior research. All questionnaire items were measured on a 7-point Likert scale ranging from “strongly disagree (1)” to “strongly agree (7)”. The questionnaire items and reference list are shown in Table 3. In addition, in order to respect the privacy of the respondents, before the questionnaire was distributed, all respondents signed an informed consent form, which clearly informed them of the voluntary nature of their participation in the study and the privacy protection measures. The entries of the questionnaire and the list of relevant references are detailed in Table 3.

**Table 3:** Questionnaire entries and references.

Variable	Item/question	References
1. Information search	1.1. The AIGC tool helps me find necessary information when I am uncertain about answers. 1.2. The AIGC tool assists me in verifying the accuracy of information. 1.3. The AIGC tool enables me to find resources related to learning and coursework, supporting my studies. 1.4. The AIGC tool provides a wealth of helpful information, aiding in a deeper understanding of various topics.	Baek and Kim, 2023; Wu et al., 2024
2. Learning needs	2.1. The AIGC tool helps me complete assignments and learning tasks, enhancing my learning outcomes. 2.2. The AIGC tool provides inspiration and generates sketches to support my creative projects. 2.3. The AIGC tool significantly aids me in writing research papers, projects, or articles. 2.4. The AIGC tool allows me to realize my ideas, overcome professional barriers, and improve learning efficiency.	Gao, 2023; Le et al., 2024
3. Enjoyment	3.1. I feel happy and entertained when using the AIGC tool. 3.2. I feel relaxed and can alleviate stress while using the AIGC tool. 3.3. I often become unaware of the passage of time while engaging with the AIGC tool. 3.4. I don't feel bored while using the AIGC tool.	Nguyen and Nguyen, 2024; Niu et al., 2024
4. Novelty	4.1. I find the AIGC tool very novel. 4.2. Compared to other tools, I find the AIGC tool unique. 4.3. I use the AIGC tool to explore the latest technological trends. 4.4. I use the AIGC tool to discover new possibilities with AI technology.	Le et al., 2024
5. Perceived usefulness	5.1. The AIGC tool effectively meets my learning needs by solving my learning challenges. 5.2. Using the AIGC tool provides me with richer learning content, enhancing my learning outcomes. 5.3. The AIGC tool is very helpful for my studies, allowing me to complete tasks more efficiently. 5.4. Using the AIGC tool has significantly improved my learning efficiency and quality.	Liao et al., 2022; Le et al., 2024
6. Sense of achievement	6.1. The AIGC tool helps me reach a higher level in my studies. 6.2. I feel my knowledge and skills are stronger than others, thanks to the AIGC tool. 6.3. The results achieved through the AIGC tool make me feel proud and boost my self-confidence. 6.4. I use the AIGC tool to gain better academic results and recognition among my peers.	Gao, 2023
7. Intelligent interaction	7.1. I believe the AIGC tool demonstrates a high level of intelligent interaction in learning. 7.2. The AIGC tool intelligently identifies and supports my various learning needs. 7.3. The AIGC tool responds to my requests in a timely and effective manner. 7.4. The intelligent interaction provided by the AIGC tool makes my learning experience smoother and more natura	Gao, 2023; Maheshwari, 2024; Wu et al., 2024
8. Convenience	8.1. The AIGC tool allows me to study conveniently anytime and anywhere. 8.2. I find the AIGC tool brings great convenience to my learning process. 8.3. Using the AIGC tool helps me save time and effort. 8.4. Compared to traditional learning methods, I find using the AIGC tool more convenient.	Gao, 2023; Huang et al., 2024; Nguyen and Nguyen, 2024
9. Social influence	9.1. My friends use the AIGC tool and encourage me to use it as well. 9.2. I believe that influential people are using the AIGC tool for their studies. 9.3. My teachers support my use of the AIGC tool to enhance learning outcomes. 9.4. My family supports my use of the AIGC tool in my studies.	Du and Lv, 2024; Ustun et al., 2024
10. Employability	10.1. I believe the skills gained through the AIGC tool enhance my employability. 10.2. I believe the AIGC tool helps me acquire key skills and competencies needed for employment. 10.3. I feel that using the AIGC tool strengthens my self-learning and continuous improvement abilities for work. 10.4. I believe using the AIGC tool keeps my skills aligned with current social demands.	Liu et al., 2023; Wang and Nah, 2024
11. Perceived risk	11.1. I worry that my personal data could be exposed while utilizing the AIGC tool. 11.2. I believe there is a data security risk with the AIGC tool, which could result in the misuse of my personal data. 11.3. I have concerns about the reliability of the AIGC tool in my learning process. 11.4. I worry that I might become overly reliant on the AIGC tool, which could affect my independent learning abilities.	Liao et al., 2022; Huang et al., 2024
12. Perceived cost	12.1. I find the subscription fee for the AIGC tool relatively high. 12.2. There may be high upgrade or additional costs associated with using the AIGC tool. 12.3. I feel that the AIGC tool demands a considerable amount of time and effort. 12.4. Overall, I believe the combined cost of using the AIGC tool is high.	Liao et al., 2022; Huang et al., 2024
13. Perceived Trust	13.1. I trust that the AIGC tool provides reliable and accurate information for my studies. 13.2. I believe the AIGC tool can effectively support my learning needs and help me acquire the necessary knowledge. 13.3. I have confidence that the AIGC tool can safeguard my personal data and reduce data leakage risks. 13.4. I believe the learning support services provided by the AIGC tool are worth the cost and do not incur excessive additional costs.	Baek and Kim, 2023; Maheshwari, 2024; Nguyen and Nguyen, 2024
14. Sustained usage intention	14.1. I intend to keep utilizing the AIGC tool to support my learning going forward. 14.2. I will prioritize the AIGC tool as a learning aid. 14.3. I am inclined to keep employing the AIGC tool throughout my studies and explore its additional features. 14.4. I would recommend my classmates and friends use the AIGC tool for learning.	Gao, 2023; Nguyen and Nguyen, 2024

### 4.2 Data collection

This study employed the Chinese online survey platform Wenjuanxing to create the questionnaire link and QR code. The questionnaire was distributed through WeChat and QQ university student groups. A snowball sampling approach was adopted, whereby initial participants were invited to forward the survey link to other university students who had experience using AIGC tools in learning-related contexts. To encourage participation, respondents who completed the questionnaire were entered into a prize draw. The target participants were Chinese university students aged 18 years or older who had experience using AIGC tools in learning-related contexts. Before completing the questionnaire, all participants were presented with an electronic informed consent statement on the first page of the online survey. The statement explained the purpose of the study, the voluntary nature of participation, anonymity, data privacy protection, and the academic use of the collected data. Only participants who agreed to participate proceeded to complete the questionnaire.

To reduce the potential limitations of snowball sampling, several measures were adopted during data collection. First, the questionnaire was distributed through more than one online channel, including WeChat and QQ university student groups, rather than relying on a single recruitment source. Second, the inclusion criteria were clearly stated at the beginning of the questionnaire to ensure that participants were university students with experience using AIGC tools in learning-related contexts. Third, participants were encouraged to share the questionnaire with eligible peers from different student networks to reduce the homogeneity of the sample.

To protect participants' anonymity and privacy, the questionnaire did not collect personally identifiable information or detailed institutional, regional, or discipline-level information, such as the specific university, region, or academic major of each respondent. In addition, the questionnaire did not ask participants to report the specific AIGC platforms or tool types they had used, because this study focused on students' general experience with AIGC tools rather than platform-specific usage. Therefore, this study reports only general demographic characteristics, including gender, age, educational level, experience with AIGC tools, and frequency of AIGC tool use.

A total of 549 responses were collected. After screening, 21 invalid responses were excluded. The exclusion criteria included incomplete questionnaires, obviously patterned or identical responses across most items, abnormal completion time, and responses containing apparent logical contradictions. Finally, 528 valid responses were retained for data analysis. Detailed demographic information is presented in Table 4.

**Table 4:** Demographic details of participants (*n* = 528).

Variable	Options	Frequency	Percentage
Gender	Male	247	46.78%
	Female	281	53.22%
Age	18–24	292	55.30%
	25–29	180	34.09%
	30 and above	56	10.61%
Education	Undergraduate	223	42.24%
	Master's	176	33.33%
	Doctoral	129	24.43%
Experience with AIGC tools	Less than 3 months	107	20.27%
	3-6 months	119	22.54%
	6–12 months	127	24.05%
	1 year or more	175	33.14%
Frequency of Using AIGC tools	At least once a day	98	18.56%
	At least once a week	192	36.36%
	At least once a month	175	33.15%
	Other	63	11.93%

## 5 Results analysis

This research utilized Smart PLS 4 for data analysis. PLS-SEM (Partial Least Squares Structural Equation Modeling) is a variance-based analytical approach well-suited for exploratory studies involving complex models and small sample sizes and is extensively applied in the social sciences (Hair et al., 2011, 2019).

### 5.1 Model fitting

This study assessed the model's suitability using the Standardized Root Mean Square Residual (SRMR) and the Normed Fit Index (NFI). The SRMR value of 0.034, well below the 0.08 threshold, indicates a strong fit with the actual data (Henseler et al., 2016). NFI measures the model's fit compared to a null model, with higher values indicating greater explanatory power (Gupta and Singharia, 2021). These indicators collectively confirm the high reliability and validity of this study's model. The model fit indices are summarized in Table 5.

**Table 5:** The results of the construct assessment.

Fit index	Computed values
SRMR	0.034
NFI	0.880

### 5.2 Common method bias

Because this study relied on self-reported questionnaire data, both procedural and statistical approaches were adopted to assess and reduce the potential influence of common method bias. In terms of procedural control, the questionnaire was anonymous, and participants were informed that there were no right or wrong answers. The items were clearly worded and adapted from established scales to reduce ambiguity and response bias.

Statistically, Harman's single-factor test was first conducted to assess common method bias. The results showed that the largest unrotated factor explained only 21.69% of the total variance, which was well below the empirical threshold of 40%. This indicates that no single factor dominated the variance structure. In addition, VIF values were calculated to examine potential collinearity problems in the structural model. As shown in Table 6, all VIF values ranged from 1.092 to 1.503, which were below the recommended threshold of 3.0 (Kim, 2019). These results suggest that common method bias was unlikely to be a major concern in this study, and the model estimation results were relatively stable.

**Table 6:** VIF values for collinearity diagnostics.

Paths	VIF
CON -> SUI	1.238
EMP -> SUI	1.197
ENJ -> SUI	1.092
INI -> SUI	1.249
INS -> SUI	1.340
LN -> SUI	1.463
NOV -> SUI	1.314
PC -> SUI	1.130
PR -> SUI	1.135
PT -> SUI	1.112
PU -> SUI	1.305
SI -> SUI	1.296
SOA -> SUI	1.503

### 5.3 Reliability and validity analysis

This study evaluated the measurement quality of the questionnaire through reliability and validity analysis. The Cronbach's Alpha and composite reliability values (rho_a and rho_c) for the questionnaire exceeded 0.7, indicating good reliability (Cronbach, 1951). Specifically, all variables had Cronbach's Alpha values above 0.8, demonstrating excellent reliability. The AVE values for all variables were greater than 0.5, reflecting good internal consistency (Bagozzi and Yi, 1988). The factor loadings for each variable exceeded 0.7, further verifying the questionnaire's convergent validity (Child, 2006). These results confirm that the questionnaire design demonstrates outstanding reliability and validity, providing reliable data support for subsequent model analysis. Detailed factor loadings and reliability indicators for each variable are presented in Table 7.

**Table 7:** Indicators of the reliability and validity of the concept.

Variable	Items	Factor loadings	α	CR (rho_a)	CR (rho_c)	AVE
Information search	INS1	0.899	0.916	0.918	0.941	0.799
	INS2	0.898				
	INS3	0.876				
	INS4	0.903				
Learning needs	LN1	0.896	0.903	0.909	0.932	0.774
	LN2	0.878				
	LN3	0.853				
	LN4	0.893				
Enjoyment	ENJ1	0.913	0.892	0.955	0.924	0.752
	ENJ2	0.830				
	ENJ3	0.855				
	ENJ4	0.869				
Novelty	Nov1	0.890	0.899	0.899	0.930	0.768
	Nov2	0.873				
	Nov3	0.857				
	Nov4	0.885				
Perceived usefulness	PU1	0.874	0.909	0.912	0.936	0.786
	PU2	0.891				
	PU3	0.883				
	PU4	0.897				
Sense of achievement	SOA1	0.886	0.901	0.908	0.931	0.770
	SOA2	0.890				
	SOA3	0.852				
	SOA4	0.881				
Intelligent interaction	INI1	0.898	0.901	0.906	0.931	0.770
	INI2	0.861				
	INI3	0.867				
	INI4	0.884				
Convenience	CON1	0.898	0.920	0.920	0.943	0.806
	CON2	0.898				
	CON3	0.896				
	CON4	0.900				
Social influence	SI1	0.874	0.895	0.897	0.927	0.761
	SI2	0.873				
	SI3	0.848				
	SI4	0.893				
Employability	EMP1	0.884	0.897	0.897	0.928	0.764
	EMP2	0.877				
	EMP3	0.858				
	EMP4	0.878				
Perceived risk	PR1	0.876	0.919	0.932	0.943	0.804
	PR2	0.909				
	PR3	0.907				
	PR4	0.894				
Perceived cost	PC1	0.893	0.908	0.931	0.935	0.782
	PC2	0.911				
	PC3	0.872				
	PC4	0.860				
Perceived trust	PT1	0.910	0.922	0.932	0.944	0.809
	PT2	0.901				
	PT3	0.904				
	PT4	0.883				
Sustained usage intention	SUI1	0.907	0.924	0.924	0.946	0.815
	SUI2	0.900				
	SUI3	0.903				
	SUI4	0.901				

The analysis results in Table 8 indicate that the standardized correlation coefficients between dimensions in the discriminant validity assessment are lower than the square roots of the corresponding AVE values. This confirms strong discriminant validity among the dimensions (Fornell and Larcker, 1981).

**Table 8:** Discriminant validity (Fornell-Larcker criterion).

Construct	CON	EMP	ENJ	INI	INS	LN	NOV	PC	PR	PT	PU	SI	SOA	SUI
CON	0.898													
EMP	0.181	0.874												
ENJ	0.186	0.108	0.867											
INI	0.181	0.215	0.127	0.878										
INS	0.176	0.219	0.072	0.268	0.894									
LN	0.308	0.246	0.145	0.291	0.292	0.880								
NOV	0.285	0.210	0.074	0.284	0.288	0.273	0.876							
PC	0.182	0.077	0.101	0.169	0.104	0.160	0.156	0.884						
PR	0.074	0.058	0.046	0.049	−0.015	0.044	0.067	0.099	0.897					
PT	0.137	0.135	0.153	0.123	0.156	0.143	0.087	0.086	0.080	0.900				
PU	0.225	0.218	0.135	0.186	0.235	0.193	0.217	0.122	0.281	0.219	0.886			
SI	0.242	0.273	0.154	0.268	0.235	0.308	0.298	0.145	0.028	0.137	0.219	0.872		
SOA	0.243	0.265	0.121	0.328	0.364	0.425	0.363	0.189	−0.024	0.110	0.188	0.341	0.878	
SUI	0.330	0.394	0.163	0.354	0.439	0.477	0.385	0.072	−0.096	0.231	0.383	0.388	0.472	0.903

According to the HTMT criterion, an HTMT value below 0.85 indicates strong discriminant validity between variables. The analysis results in Table 9 reveal that the heterotrait-monotrait ratio (HTMT) for all traits in this study remains below 0.85. These findings further confirm that the model in this study exhibits good discriminant validity (Fornell and Larcker, 1981).

**Table 9:** Discriminant validity (HTMT values).

Construct	CON	EMP	ENJ	INI	INS	LN	NOV	PC	PR	PT	PU	SI	SOA	SUI
CON														
EMP	0.200													
ENJ	0.197	0.112												
INI	0.198	0.237	0.132											
INS	0.192	0.241	0.075	0.294										
LN	0.337	0.272	0.145	0.320	0.319									
NOV	0.314	0.233	0.088	0.317	0.317	0.301								
PC	0.196	0.089	0.105	0.186	0.113	0.177	0.170							
PR	0.086	0.069	0.055	0.058	0.039	0.050	0.079	0.110						
PT	0.148	0.148	0.164	0.130	0.168	0.155	0.097	0.095	0.094					
PU	0.246	0.240	0.143	0.203	0.258	0.213	0.239	0.129	0.313	0.240				
SI	0.266	0.305	0.162	0.301	0.258	0.340	0.332	0.165	0.035	0.150	0.241			
SOA	0.265	0.292	0.122	0.362	0.399	0.468	0.402	0.208	0.042	0.118	0.205	0.377		
SUI	0.358	0.432	0.169	0.386	0.477	0.519	0.423	0.077	0.103	0.247	0.417	0.426	0.513	

### 5.4 Hypothesis testing

This study used SmartPLS 4 to conduct PLS-SEM analysis. All constructs in the measurement model were specified as reflective constructs because the measurement items were designed to reflect the underlying latent variables. The significance of the path coefficients was assessed using a bootstrapping procedure with 5,000 subsamples. A two-tailed test was applied at the 0.05 significance level, and 95% confidence intervals were used to evaluate the significance of the hypothesized relationships (Mulaik et al., 1989).

This study assessed the path coefficients (β), coefficient of determination (*R*^2^), predictive relevance (*Q*^2^), and effect size (*f*^2^) of the structural model. The PLS algorithm, bootstrapping, and blindfolding procedures were used to evaluate the explanatory and predictive quality of the model. Regarding explanatory power, the *R*^2^ value of sustained usage intention was 0.558, and the adjusted *R*^2^ value was 0.543. This indicates that the proposed model explained 55.8% of the variance in students' sustained usage intention toward AIGC tools, suggesting a moderate level of explanatory power. In terms of predictive relevance, the *Q*^2^ value obtained through the blindfolding procedure was used to evaluate the model's predictive relevance. According to commonly used criteria, Q^2^ values of 0.02, 0.15, and 0.35 indicate small, medium, and large levels of predictive relevance, respectively. In this study, the *Q*^2^ value of sustained usage intention was 0.443, indicating that the model had strong predictive relevance. In addition, the effect size *f*^2^ was used to further assess the relative contribution of each predictor. The results showed that perceived usefulness had the largest effect size on sustained usage intention (*f*^2^ = 0.079), followed by perceived risk (*f*^2^ = 0.068), learning needs (*f*^2^ = 0.061), information search (*f*^2^ = 0.043), employability (*f*^2^ = 0.039), sense of achievement (*f*^2^ = 0.031), and perceived cost (*f*^2^ = 0.021). Overall, these results suggest that the model demonstrated acceptable explanatory power and good predictive relevance as an integrated framework.

The results indicated that several factors were significantly and positively associated with students' sustained usage intention toward AIGC tools. Specifically, information search (β = 0.160, *p* < 0.001), learning needs (β = 0.198, *p* < 0.001), novelty (β = 0.100, *p* ≤ 0.01), perceived usefulness (β = 0.213, *p* < 0.001), sense of achievement (β = 0.142, *p* ≤ 0.001), intelligent interaction (β = 0.084, *p* < 0.05), convenience (β = 0.077, *p* < 0.05), social influence (β = 0.092, *p* < 0.05), and employability (β = 0.144, *p* < 0.001) were all positively associated with sustained usage intention. These results support hypotheses H1a, H1b, H2b, H3a, H3b, H4a, H4b, H5a, and H5b. In particular, perceived usefulness showed the strongest positive association with sustained usage intention, suggesting that students' perceived practical value of AIGC tools is closely related to their willingness to continue using them.

However, enjoyment was not significantly associated with students' sustained usage intention toward AIGC tools (β = 0.013, *p* > 0.05), so hypothesis H2a was not supported. This finding suggests that enjoyment may not be a key factor associated with students' continued use of AIGC tools in educational contexts.

**Figure 2:**
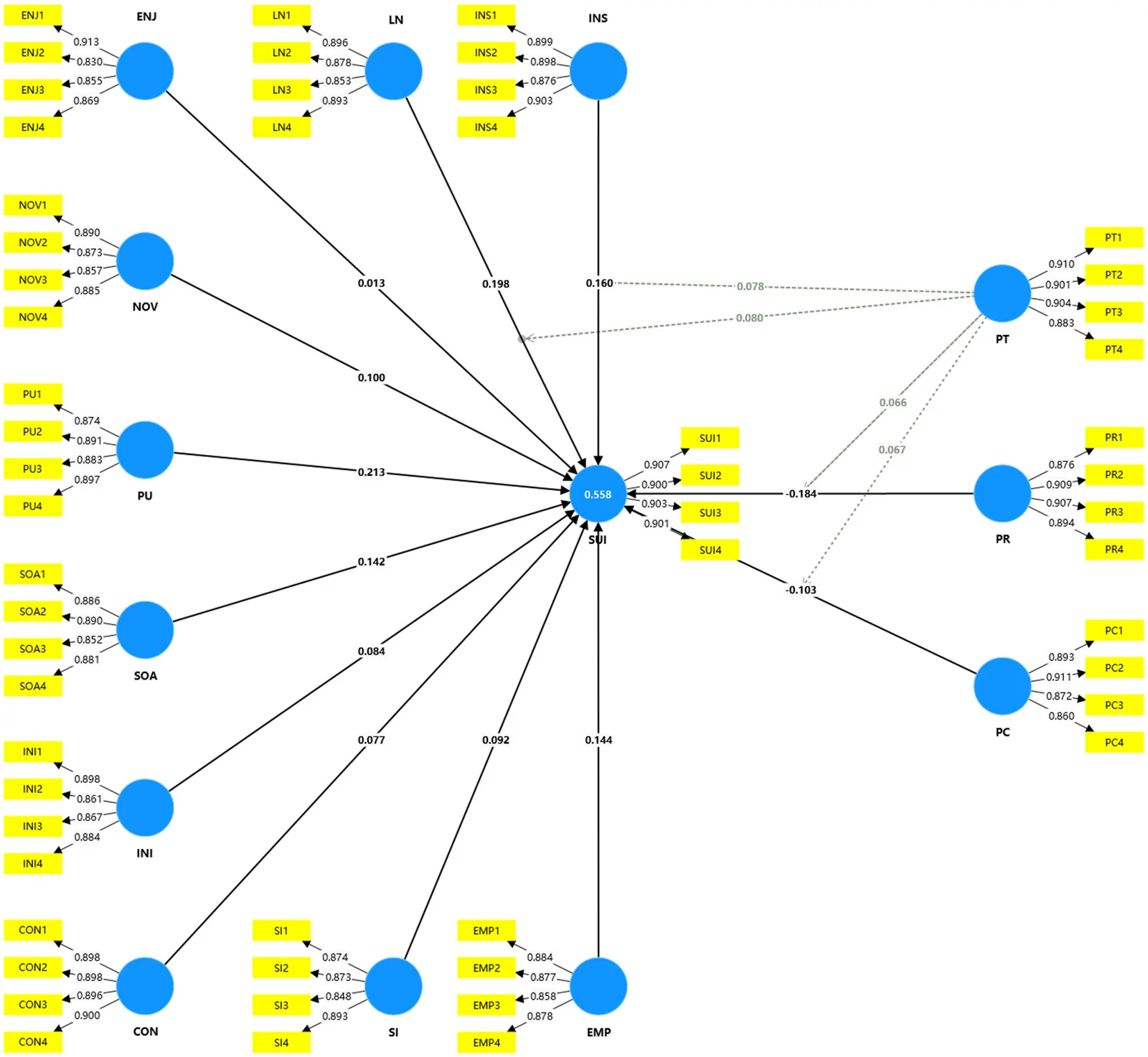
Results of PLS structural model.

In terms of negative factors, perceived risk and perceived cost were significantly and negatively associated with students' sustained usage intention toward AIGC tools. Perceived risk (β = −0.184, *p* < 0.001) showed the strongest negative association, followed by perceived cost (β = −0.103, *p* < 0.05). These results support hypotheses H6a and H6b, suggesting that students' concerns about potential risks and costs are negatively related to their willingness to continue using AIGC tools. The detailed path estimates are summarized in Table 10, and the results of the PLS structural model are presented in Figure 2.

**Table 10:** Analysis of pathway relationships.

Paths	β	*t*-value	*p*-Value	2.50%	97.50%	Results
H1a:INS -> SUI	0.160	3.829	0.000	0.079	0.243	Supported
H1b: LN -> SUI	0.198	4.425	0.000	0.112	0.286	Supported
H2a: ENJ -> SUI	0.013	0.391	0.696	−0.051	0.077	Unsupported
H2b: NOV-> SUI	0.100	2.585	0.010	0.026	0.179	Supported
H3a: PU -> SUI	0.213	5.722	0.000	0.142	0.287	Supported
H3b: SOA -> SUI	0.142	3.216	0.001	0.054	0.226	Supported
H4a:INI->SUI	0.084	2.250	0.024	0.012	0.159	Supported
H4b: CON -> SUI	0.077	2.056	0.040	0.005	0.152	Supported
H5a:SI -> SUI	0.092	2.408	0.016	0.017	0.167	Supported
H5b: EMP -> SUI	0.144	3.966	0.000	0.073	0.216	Supported
H6a:PR -> SUI	−0.184	6.053	0.000	−0.239	−0.126	Supported
H6b: PC -> SUI	−0.103	3.237	0.001	−0.157	−0.032	Supported

### 5.5 Moderation analysis

In this study, we examined the moderating role of perceived trust on the relationships between variables (Ponte et al., 2015). The results showed that perceived trust significantly strengthened the relationship between information search and sustained usage intention (H7a: β = 0.078, *p* < 0.05), as well as the relationship between learning needs and sustained usage intention (H7b: β = 0.080, *p* < 0.05). These findings indicate that when students have higher trust in AIGC tools, the positive effects of information search and learning needs on sustained usage intention become stronger.

The results also showed that perceived trust significantly moderated the relationship between perceived risk and sustained usage intention (H7c: β = 0.066, *p* < 0.05), as well as the relationship between perceived cost and sustained usage intention (H7d: β = 0.067, *p* < 0.05). These findings suggest that higher perceived trust can weaken the negative effects of perceived risk and perceived cost on students' sustained usage intention. In other words, when students trust the reliability, usefulness, and data protection of AIGC tools, their concerns about risks and costs are less likely to reduce their willingness to sustain use.

The interaction plots are provided in Supplementary Figures S1–S4 to further illustrate the moderating effects of perceived trust. The moderation results are summarized in Table 11.

**Table 11:** Results of moderation.

*H*	Relationship	β	*T*-value	*P*	2.50%	97.50%	Supported
H7a	PT x INS -> SUI	0.078	2.115	0.034	0.005	0.149	Yes
H7b	PT x LN -> SUI	0.080	2.234	0.026	0.006	0.148	Yes
H7c	PT x PR -> SUI	0.066	2.219	0.027	0.005	0.12	Yes
H7d	PT x PC -> SUI	0.067	2.026	0.043	−0.002	0.129	Yes

### 5.6 Multi-group analysis

The purpose of the multi-group analysis (MGA) is to identify variations across user segments distinguished by demographic attributes. Prior research has emphasized that factors such as gender, age, and educational attainment often serve as crucial moderators influencing behavioral differences (Liu et al., 2025). Detailed profiles of each subgroup are summarized in Table 12.

**Table 12:** Subgroup profiles for multi-group analysis.

Group	Subgroup	Group size
Gender	Male	247
	Female	281
Age	Young (age ≤ 24)	292
	Old (age ≥ 25)	236
Education	Undergraduate	223
	Postgraduate (Master's and Doctoral)	305
Experience with AIGC tools	Within 6 months	226
	More than 6 months	302
Frequency of using AIGC tools	High (≥ 1 time per week)	290
	Low ( ≤ 1 time per month)	238

Prior to performing the MGA, it is essential to verify measurement invariance as a preliminary condition. Following previous studies and methodological guideline (Wu et al., 2023), this study employed the Measurement Invariance of Composite Models procedure to sequentially test configural invariance, compositional invariance, and the equality of means and variances. Using the permutation algorithm in PLS-SEM, measurement invariance was confirmed (see Supplementary Table S1), providing a solid foundation for the subsequent MGA. The results indicated that all major demographic variables satisfied the measurement invariance requirements.

The results of the MGA (Table 13) indicate significant differences among different groups in their use of AI tools. Specifically, in terms of education level, the effect of Information Search on continuance intention is more pronounced among postgraduate students, while the moderating role of trust in the relationship between Learning Needs and continuance intention is stronger among undergraduate students. Regarding usage frequency, the effect of Novelty on continuance intention is significant among low-frequency users but not among high-frequency users. In contrast, no significant differences were observed across gender, age, or usage experience.

**Table 13:** MGA results.

Group	Relationship	β0	β1	Coefficien*t* difference
Undergraduate vs postgraduate	INS -> SUI	0.088	0.258^***^	−0.17^*^
	PT x LN -> SUI	0.189^**^	−0.042	0.231^**^
High frequency vs low frequency	NOV -> SUI	0.003	0.185^**^	−0.182^*^

^***^p < 0.001; ^**^p < 0.01; ^*^p < 0.05.

## 6 Discussion

This study integrates UGT and PVT to examine factors influencing students' consistent use of AIGC tools and the moderating role of perceived trust. The results showed that different types of satisfaction significantly drive continued use, while perceived risk and cost are key barriers. Additionally, perceived trust strengthens the positive impact of factors and reduces the negative effects of perceived risk and cost.

### 6.1 Uses and Gratifications Theory and the influence of positive factors

The results of this study confirm the influence of five dimensions of gratification on students' continuance intention to use AIGC tools. Among them, content gratification and utilitarian gratification exert the most significant effects, followed by social gratification, while the impacts of hedonic gratification and technical gratification are relatively weaker but still statistically significant. This hierarchical relationship reveals the core needs and primary considerations of students when using AIGC tools, providing important insights for the educational integration, instructional design, and optimization of AIGC tools in higher education.

First, the positive influence of the two dimensions of content gratification, H1a Information Search and H1b Learning Need, on sustained usage intention was verified. This is consistent with the findings of Gao (2023) on intelligent mobile learning applications and Baek and Kim (2023) on ChatGPT. In particular, the significance of learning needs reflects the key role of AIGC tools in meeting students' personalized learning goals and enhancing the learning experience. It is recommended that universities and educators collaborate with AIGC tool developers to design discipline-specific teaching scenarios, learning tasks, and instructional support mechanisms, so as to improve the applicability and educational impact of AIGC tools in higher education.

Second, among utilitarian gratification, H3a perceived usefulness had the most significant effect on sustained usage intention. This result is consistent with (Jo H. 2023) research on smart factories and Kim and Song (2022) research on MOOCs. This indicates that students are more concerned about the practical value of AIGC tools in learning, such as improving learning efficiency and helping to achieve specific goals. At the same time, H3b sense of achievement also significantly promoted students' sustained usage intention. This is consistent with the study by Gao (2023), which shows that the sense of achievement obtained after completing a learning task serves as a key motivator in sustaining the use of the tool. Therefore, universities, educators, and educational technology developers should jointly focus on optimizing the learning-support functions of AIGC tools, such as accurate knowledge recommendations, personalized learning paths, task-based guidance, and real-time feedback mechanisms, to further meet students' utilitarian learning needs.

Third, in terms of social gratification, both H5a social influence and H5b employability significantly affect students' sustained usage intention. The results of social influence show that students' behavior is largely influenced by the approval and usage behavior of the surrounding group. This result is consistent with the findings of (Biloš and Budimir 2024) and Sobaih et al. (2024) on ChatGPT research. Especially in educational settings, teacher and peer recommendations or general usage can significantly increase student acceptance of the tool. It is recommended that educators guide students to use AIGC tools in collaborative learning, peer discussion, project-based assignments, and classroom activities, while developers may provide team collaboration functions to enhance interaction among students. The significant impact of employability further emphasizes the value of AIGC tools in helping students improve their vocational skills and competitiveness. This aligns with the research of Liu et al. (2023), which shows that students expect the tool to help them acquire more industry-relevant skills. It is recommended that universities and educators embed AIGC tools into career-oriented learning activities, such as skills training, project practice, industry trend analysis, and career planning tasks, while developers may provide functions that support students' long-term skill development and employability.

In addition, in terms of hedonic gratification, the significant positive effect of H2b Novelty on sustained usage intention indicates that innovation and uniqueness are important drivers in motivating students to use AIGC tools. This result aligns with the study by Bui et al. (2024) study on AI teaching assistants. Universities, educators, and AIGC tool developers can jointly update learning scenarios, instructional resources, and tool functions to maintain students' interest while ensuring that novelty serves learning objectives rather than entertainment alone. However, the influence of H2a enjoyment on continued use intention did not reach a significant level, which may reflect that students value the practical functions of the AIGC tool in educational scenarios more than its entertainment value. Similar research such as Wu et al. (2024) on the Metaverse Museum also reached a similar conclusion.

Finally, the positive influence of the two dimensions of technology gratification, H4a intelligent interaction and H4b convenience, on sustained usage intention further illustrates the importance of interactive experience and operational convenience. Consistent with the findings of Moussawi et al. (2023), it is advised to refine the user interface design, simplify the operation process, and enhance intelligent interactive functions so that AIGC tools can be more easily embedded into everyday learning tasks, classroom activities, and independent study.

### 6.2 Theory of perceived value and the influence of negative factors

The analysis of negative factors shows that perceived risk and perceived cost significantly inhibit students' sustained usage intention. Among them, the impact of Perceived risk is the most significant, reflecting students' strong worries regarding privacy violations, data security, and technical reliability matters. This outcome aligns with the research conducted by Dogra and Kaushal (2021). To reduce perceived risk, universities and developers should jointly establish data protection mechanisms, transparent privacy policies, clear usage boundaries, and regular security reviews. Educators should also guide students to critically evaluate AI-generated content and avoid overdependence on AIGC tools in learning tasks.

The negative impact of perceived cost was also verified, indicating that students' concerns about the high economic, time, and energy investment in tools can significantly reduce their acceptance. This finding is consistent with the research of Twum et al. (2022). To reduce perceived cost, universities and developers may provide institutional access, free trials, low-cost educational versions, or modular functions tailored to different learning needs. In addition, educators can help reduce students' time and effort costs by offering clear instructions and examples of how to use AIGC tools effectively in learning activities.

### 6.3 The moderating effect of perceived trust

Perceived trust played a significant role as an important moderating variable between positive and negative factors. The results showed that a high level of perceived trust enhanced the positive impact of positive factors (such as information search and learning needs) and weakened the negative impact of negative factors (such as perceived risk and perceived cost). This finding is consistent with the results of Yousafzai et al. (2003).

Specifically, when perceived trust is high, students are more willing to ignore possible risks and cost barriers, and focus more on the practical value of the tool in terms of learning goals and career development. Therefore, enhancing user trust is crucial for the continued use of the tool. It is recommended that universities, educators, and developers jointly build students' perceived trust by providing transparent functional demonstrations, clear operation guides, examples of responsible use, and institutional endorsement. In teaching practice, educators should explain the strengths and limitations of AIGC tools, guide students to verify AI-generated content, and clarify ethical boundaries related to academic integrity, privacy, and data security.

### 6.4 MGA discussion

The multi-group analysis results further reveal significant differences among different groups in their use of AIGC tools. First, regarding educational level, the influence of information search on continuance intention is more pronounced among postgraduate students, whereas trust plays a stronger moderating role between learning needs and continuance intention among undergraduate students. This may be because postgraduate students possess stronger autonomous learning capacity and analytical thinking skills, enabling them to independently evaluate and filter valuable information in complex informational environments. As a result, they tend to recognize the practical benefits of AIGC tools in information retrieval and knowledge integration, thereby enhancing their motivation for continued use. In contrast, undergraduate students, with relatively limited cognitive experience and academic training, may lack sufficient ability to evaluate the authenticity, precision, as well as the applicability of AI-generated content. Consequently, they tend to rely more on trust in the system to reduce uncertainty during use. In this context, trust serves as a psychological safety mechanism which helps them overcome concerns and unfamiliarity with AI tools, thus maintaining a higher level of usage intention.

Second, in terms of usage frequency, the effect of novelty on continuance intention is stronger among low-frequency users but insignificant among high-frequency users. This suggests that for low-frequency or first-time users, the novelty and exploratory experience brought by the technology serve an important function in motivating their willingness to use. However, as usage frequency increases, users gradually shift to a more rational usage stage, focusing more on the tool's functional improvement and learning effectiveness, while the emotional impact of novelty weakens.

In addition, gender, age, and length of usage experience do not show significant differences, indicating that these demographic factors have relatively weak effects on continuance intention. The psychological mechanisms underlying students' usage behavior are more strongly influenced by their educational background and usage habits.

These group differences suggest that the integration of AIGC tools into higher education should not adopt a one-size-fits-all approach. For postgraduate students, universities may emphasize advanced information retrieval, knowledge integration, and research support. For undergraduate students, educators should provide more guidance, trust-building mechanisms, and examples of responsible use. For low-frequency users, introductory training and exploratory learning activities may help reduce unfamiliarity and stimulate initial engagement, whereas high-frequency users may benefit more from advanced functions, efficiency improvement, and discipline-specific applications.

### 6.5 Theoretical and practical significance

From a theoretical perspective, this study provides an integrated explanation of students' sustained usage intention toward AIGC tools in educational settings. Unlike traditional technology acceptance studies that mainly emphasize usefulness, ease of use, or social influence, this study adopts UGT and PVT as the main theoretical framework to examine both positive gratifications and perceived sacrifices. Specifically, UGT explains the content, hedonic, utilitarian, technological, and social gratifications that students may obtain from AIGC tool use, while PVT helps explain how perceived risk and perceived cost may reduce their willingness to continue using these tools. In this sense, the study moves beyond a purely acceptance-oriented perspective and offers a more balanced understanding of students' psychological evaluation of AIGC tools. In addition, by introducing perceived trust as a moderating variable, this study further clarifies the boundary condition under which gratifications and sacrifices are associated with sustained usage intention. These findings enrich the application of UGT and PVT in AI-supported learning contexts and provide a theoretical basis for understanding students' continued use of AIGC tools.

From a practical perspective, the findings provide useful references for universities, educators, and AIGC tool developers. For universities and educators, the positive associations of perceived usefulness, learning needs, and information search with sustained usage intention suggest that AIGC tools should be introduced in ways that directly support students' learning tasks, such as information retrieval, assignment completion, academic writing, creative ideation, and problem solving. Educators may guide students to use these tools as learning-support resources and help them evaluate, verify, and refine AI-generated content.

The negative associations of perceived risk and perceived cost suggest that universities should pay attention to students' concerns about privacy, data security, reliability, overdependence, and financial cost. Clear usage guidelines, privacy protection measures, academic integrity rules, and transparent information about tool access may help reduce students' uncertainty. Moreover, the moderating role of perceived trust indicates the importance of strengthening students' trust in the reliability, usefulness, and data protection of AIGC tools through classroom guidance, case analysis, and critical discussion.

For AIGC tool developers, the findings suggest that improving functional usefulness alone may not be sufficient. Developers should also enhance information accuracy, interaction quality, privacy protection, cost transparency, and learning-oriented feedback functions to better support students' responsible use of AIGC tools in educational contexts.

## 7 Conclusion

This study examined the psychological factors associated with students' sustained usage intention toward AIGC tools in educational settings by integrating the Uses and Gratifications Theory and the Perceived Value Theory. Based on questionnaire data from 528 Chinese university students and PLS-SEM analysis, the findings show that learning needs, perceived usefulness, information search, employability, sense of achievement, novelty, intelligent interaction, convenience, and social influence are significantly and positively associated with students' sustained usage intention toward AIGC tools. Among these factors, learning needs and perceived usefulness show the strongest positive associations, indicating that students are more likely to continue using AIGC tools when they perceive them as useful for learning tasks, information acquisition, academic support, and future career development. However, enjoyment is not significantly associated with continued use, suggesting that students' sustained use of AIGC tools in educational contexts may be more closely related to practical learning value than to entertainment-oriented experience.

The results also reveal that perceived risk and perceived cost are significantly and negatively associated with students' sustained usage intention, reflecting their concerns about privacy, data security, system reliability, overdependence, financial cost, and time investment. Perceived trust plays an important moderating role by strengthening the positive associations of information search and learning needs with sustained usage intention while weakening the negative associations of perceived risk and perceived cost with sustained usage intention. In addition, multi-group analysis indicates that different student groups show distinct usage mechanisms: postgraduate students place greater emphasis on information retrieval, undergraduate students rely more on trust to reduce uncertainty, and infrequent users are more strongly associated with novelty.

Overall, this study suggests that students' sustained usage intention toward AIGC tools in higher education is associated not only with technological functionality, but also with pedagogical integration, trust-building, risk governance, cost concerns, and differentiated instructional support. These findings provide practical references for universities, educators, and AIGC tool developers seeking to support the responsible, sustainable, and student-centered use of AIGC tools in teaching and learning. Future research may further examine the long-term relationships between AIGC tool use and students' learning outcomes, digital literacy, academic integrity, and career development across different educational contexts.

## 8 Limitations and future research directions

While this study provides meaningful perspectives on students' sustained usage intention toward AIGC tools, several limitations remain. First, the sample data were drawn from Chinese higher education students and collected through online university student groups using a snowball sampling approach. Although this method was suitable for reaching students with experience using AIGC tools, future studies could expand the sample to other countries, educational backgrounds, and disciplinary contexts, and adopt more diverse sampling strategies to enhance the broader applicability of the findings. Second, this study mainly relied on cross-sectional questionnaire data and examined sustained usage intention rather than actual long-term usage behavior. Therefore, the relationships identified in this study should be interpreted as predictive associations, and future research could further verify these relationships through experimental studies, longitudinal data, or behavioral tracking data. In addition, this study focused on students' general experience with AIGC tools and did not collect detailed information about specific AIGC platforms, tool types, universities, regions, or individual academic majors. Future studies could collect non-identifiable background information and explore other possible moderating variables, including technology adoption factors or cultural differences, to further improve the theoretical framework of AIGC tool usage behavior.

## Data Availability

The original contributions presented in the study are included in the article/Supplementary material, further inquiries can be directed to the corresponding author/s.

## References

[B1] Adapa A. Nah F. F.-H. Hall R. H. Siau K. Smith S. N. (2018). Factors influencing the adoption of smart wearable devices. Int. J. Hum.-Comput. Interact. 34, 399–409. doi: 10.1080/10447318.2017.1357902

[B2] Ahmad S. F. Han H. Alam M. M. Rehmat M. Irshad M. Arraño-Muñoz M. . (2023). Retracted article: impact of artificial intelligence on human loss in decision making, laziness and safety in education. Humanit. Soc. Sci. Commun. 10:311. doi: 10.1057/s41599-023-01787-8 37325188 PMC10251321

[B3] Akdim K. Casaló L. V. Flavián C. (2022). The role of utilitarian and hedonic aspects in the continuance intention to use social mobile apps. J. Retail. Consum. Serv. 66:102888. doi: 10.1016/j.jretconser.2021.102888

[B4] Alasadi E. A. Baiz C. R. (2023). Generative AI in education and research: opportunities, concerns, and solutions. J. Chem. Educ. 100, 2965–2971. doi: 10.1021/acs.jchemed.3c00323

[B5] Alimamy S. Gnoth J. (2022). I want it my way! the effect of perceptions of personalization through augmented reality and online shopping on customer intentions to co-create value. Comput. Hum. Behav. 128:107105. doi: 10.1016/j.chb.2021.107105

[B6] Al-Saedi K. Al-Emran M. Ramayah T. Abusham E. (2020). Developing a general extended UTAUT model for M-payment adoption. Technol. Soc. 62:101293. doi: 10.1016/j.techsoc.2020.101293

[B7] Al-Shamaileh O. Sutcliffe A. (2023). Why people choose apps: an evaluation of the ecology and user experience of mobile applications. Int. J. Hum. Comput. Stud. 170:102965. doi: 10.1016/j.ijhcs.2022.102965

[B8] Alzougool B. (2018). The impact of motives for facebook use on facebook addiction among ordinary users in Jordan. Int. J. Soc. Psychiatry 64, 528–535. doi: 10.1177/0020764018784616 29939103

[B9] Alzyoud M. Al-Shanableh N. Alomar S. AsadAlnaser A. Mustafad A. Al-Momani A. . (2024). Artificial intelligence in Jordanian education: assessing acceptance via perceived cybersecurity, novelty value, and perceived trust. Int. J. Data Netw. Sci. 8, 823–834. doi: 10.5267/j.ijdns.2023.12.022

[B10] Antón C. Camarero C. Rodríguez J. (2017). Pleasure in the use of new technologies: the case of e-book readers. Online Inf. Rev. 41, 219–234. doi: 10.1108/OIR-10-2015-0331

[B11] Arslan I. K. (2020). The importance of creating customer loyalty in achieving sustainable competitive advantage. Eurasian J. Bus. Manag. 8, 11–20. doi: 10.15604/ejbm.2020.08.01.002

[B12] Baek T. H. Kim M. (2023). Is ChatGPT scary good? How user motivations affect creepiness and trust in generative artificial intelligence. Telemat. Inform. 83:102030. doi: 10.1016/j.tele.2023.102030

[B13] Bagozzi R. P. Yi Y. (1988). On the evaluation of structural equation models. J. Acad. Mark. Sci. 16, 74–94. doi: 10.1007/BF02723327

[B14] Bansah A. K. Darko Agyei D. (2022). Perceived convenience, usefulness, effectiveness and user acceptance of information technology: evaluating students' experiences of a learning management system. Technol. Pedagogy Educ. 31, 431–449. doi: 10.1080/1475939X.2022.2027267

[B15] Bauer R. A. (1967). Consumer behavior as risk taking. Mark. Crit. Perspect. Bus. Manag. 593, 13–21.

[B16] Baytak A. (2023). The acceptance and diffusion of generative artificial intelligence in education: a literature review. Curr. Perspect. Educ. Res. 6, 7–18. doi: 10.46303/cuper.2023.2

[B17] Bell R. Bell H. (2023). Entrepreneurship education in the era of generative artificial intelligence. Entrep. Educ. 6, 229–244. doi: 10.1007/s41959-023-00099-x

[B18] Bhattacherjee A. (2001). Understanding information systems continuance: an expectation-confirmation model. MIS Q. 25, 351–370. doi: 10.2307/3250921

[B19] Biloš A. Budimir B. (2024). Understanding the adoption dynamics of chatgpt among generation z: insights from a modified UTAUT2 model. J. Theor. Appl. Electron. Commer. Res. 19, 863–879. doi: 10.3390/jtaer19020045

[B20] Blokker R. Akkermans J. Tims M. Jansen P. Khapova S. (2019). Building a sustainable start: the role of career competencies, career success, and career shocks in young professionals' employability. J. Vocat. Behav. 112, 172–184. doi: 10.1016/j.jvb.2019.02.013

[B21] Bui M. T. Nguyen T. P. Phan B. T. (2024). “How Students Interact with AI-Teaching Assistant: Roles of Novelty and Innovativeness,” in 11th international conference on emerging challenges: smart business and digital economy 2023 (icech 2023), (Dordrecht: Atlantis Press), 425–433. doi: 10.2991/978-94-6463-348-1_32

[B22] Cao Y. Li S. Liu Y. Yan Z. Dai Y. Yu P. S. . (2023). A Comprehensive Survey of AI-Generated Content (AIGC): A History of Generative AI from GAN to ChatGPT. Ithaca, NY: arXiv.

[B23] Celik I. (2023). Towards Intelligent-TPACK: an empirical study on teachers' professional knowledge to ethically integrate artificial intelligence (AI)-based tools into education. Comput. Hum. Behav. 138:107468. doi: 10.1016/j.chb.2022.107468

[B24] Cetinic E. She J. (2022). Understanding and creating art with AI: review and outlook. ACM Trans. Multimedia Comput. Commun. Appl. 18, 1–22. doi: 10.1145/3475799

[B25] Chang Y. Lee S. Wong S. F. Jeong S. (2022). AI-powered learning application use and gratification: an integrative model. Inf. Technol. People 35, 2115–2139. doi: 10.1108/ITP-09-2020-0632

[B26] Chao C.-Y. Chang T.-C. Wu H.-C. Lin Y.-S. Chen P.-C. (2016). The interrelationship between intelligent agents' characteristics and users' intention in a search engine by making beliefs and perceived risks mediators. Comput. Human Behav. 64, 117–125. doi: 10.1016/j.chb.2016.06.031

[B27] Chen C. Wu Z. Lai Y. Ou W. Liao T. Zheng Z. (2023). Challenges and Remedies to Privacy and Security in AIGC: Exploring the Potential of Privacy Computing, Blockchain, and Beyond. Boston, MA: Pearson Education.

[B28] Chen M. Tworek J. Jun H. Yuan Q. Pinto H. P. de O. . (2021). Evaluating Large Language Models Trained on Code. Ithaca, NY: arXiv.

[B29] Chen X. Hu Z. Wang C. (2024). Empowering education development through AIGC: a systematic literature review. Educ. Inf. Technol. 29, 17485–17537. doi: 10.1007/s10639-024-12549-7

[B30] Cheng X. Zhang X. Cohen J. Mou J. (2022). Human vs. AI: understanding the impact of anthropomorphism on consumer response to chatbots from the perspective of trust and relationship norms. Inf. Process. Manag. 59:102940. doi: 10.1016/j.ipm.2022.102940

[B31] Cheng Y. Wang Y. Zhao W. (2022). Shared virtual reality experiences during the COVID-19 pandemic: exploring the gratifications and effects of engagement with immersive videos. Int. J. Environ. Res. Public Health 19:5056. doi: 10.3390/ijerph19095056 35564451 PMC9100784

[B32] Child D. (2006). The essentials of factor analysis. A&C Black. Available online at: https://www.google.com/books?hl=zh-CN&lr=&id=rQ2vdJgohH0C&oi=fnd&pg=PR7&dq=Child,+D.+(2006).+The+essentials+of+factor+analysis+(3rd+ed.).+Continuum+International+Publishing+Group.&ots=mZ2oINjM-K&sig=9mA9dueh_BKim21oNNz_oqb-Vgw (Accessed August 20, 2024).

[B33] Choung H. David P. Ross A. (2023). Trust in AI and its role in the acceptance of AI technologies. Int. J. Hum.-Comput. Interact. 39, 1727–1739. doi: 10.1080/10447318.2022.2050543

[B34] Chunxiang L. (2014). Study on mobile commerce customer based on value adoption. J. Appl. Sci. 14, 901–909. doi: 10.3923/jas.2014.901.909

[B35] Cooper G. (2023). Examining science education in chatgpt: an exploratory study of generative artificial intelligence. J. Sci. Educ. Technol. 32, 444–452. doi: 10.1007/s10956-023-10039-y

[B36] Cronbach L. J. (1951). Coefficient alpha and the internal structure of tests. Psychometrika 16, 297–334. doi: 10.1007/BF02310555

[B37] De Vos A. Van der Heijden B. I. Akkermans J. (2020). Sustainable careers: toward a conceptual model. J. Vocat. Behav. 117:103196. doi: 10.1016/j.jvb.2018.06.011

[B38] Desha C. J. Hargroves K. Smith M. H. (2009). Addressing the time lag dilemma in curriculum renewal toward engineering education for sustainable development. Int. J. Sustain. High. Educ. 10, 184–199. doi: 10.1108/14676370910949356

[B39] Dogra P. Kaushal A. (2021). An investigation of indian generation z adoption of the voice-based assistants (VBA). J. Promot. Manag. 27, 673–696. doi: 10.1080/10496491.2021.1880519

[B40] Du L. Lv B. (2024). Factors influencing students' acceptance and use generative artificial intelligence in elementary education: an expansion of the UTAUT model. Educ. Inf. Technol. 29, 24715–24734. doi: 10.1007/s10639-024-12835-4

[B41] ElSayary A. (2024). An investigation of teachers' perceptions of using ChatGPT as a supporting tool for teaching and learning in the digital era. Comput. Assist. Learn. 40, 931–945. doi: 10.1111/jcal.12926

[B42] Essel R. E. (2022). Assessing the moderating role of trialability and perceived risk of e-banking adoption in an emerging economy. Vision. doi: 10.1177/09722629221106260. [Epub ahead of print].

[B43] Farrelly T. Baker N. (2023). Generative artificial intelligence: implications and considerations for higher education practice. Educ. Sci. 13:1109. doi: 10.3390/educsci13111109

[B44] Farrokhnia M. Banihashem S. K. Noroozi O. Wals A. (2024). A SWOT analysis of ChatGPT: Implications for educational practice and research. Innov. Educ. Teach. Int. 61, 460–474. doi: 10.1080/14703297.2023.2195846

[B45] Flanagin A. Metzger M. (2006). Internet use in the contemporary media environment. Hum. Commun. Res. 27, 153–181. doi: 10.1111/j.1468-2958.2001.tb00779.x

[B46] Fornell C. Larcker D. F. (1981). Evaluating structural equation models with unobservable variables and measurement error. J. Mark. Res. 18, 39–50. doi: 10.1177/002224378101800104

[B47] Frenkel M. E. Emara H. (2024). ChatGPT-3.5 and−4.0 and mechanical engineering: examining performance on the fe mechanical engineering and undergraduate exams. Comput. Appl. Eng. 32:e22781. doi: 10.1002/cae.22781

[B48] Gallego M. D. Bueno S. Noyes J. (2016). Second Life adoption in education: a motivational model based on uses and gratifications theory. Comput. Educ. 100, 81–93. doi: 10.1016/j.compedu.2016.05.001

[B49] Gamage T. C. Tajeddini K. Tajeddini O. (2022). Why Chinese travelers use wechat to make hotel choice decisions: a uses and gratifications theory perspective. J. Glob. Scholars Mark. Sci. 32, 285–312. doi: 10.1080/21639159.2021.1961599

[B50] Gao B. (2023). A uses and gratifications approach to examining users' continuance intention toward smart mobile learning. Humanit. Soc. Sci. Commun. 10, 1–13. doi: 10.1057/s41599-023-02239-z

[B51] G. C S. B. Bhandari P. Gurung S. K. Srivastava E. Ojha D. . (2024). Examining the role of social influence, learning value and habit on students' intention to use chatGPT: the moderating effect of information accuracy in the UTAUT2 model. Cogent Educ. 11:2403287. doi: 10.1080/2331186X.2024.2403287

[B52] Gefen D. Straub D. W. (2004). Consumer trust in B2C e-commerce and the importance of social presence: experiments in e-products and e-services. Omega 32, 407–424. doi: 10.1016/j.omega.2004.01.006

[B53] Geng L. Li Y. Zhang Y. Jiang Z. Xue Y. (2024). Advancing tourism recovery through virtual tourism marketing: an integrated approach of uses and gratifications theory and attachment to VR. Curr. Issues Tour. 27, 234–250. doi: 10.1080/13683500.2023.2177834

[B54] George A. S. George A. H. (2023). A review of ChatGPT AI's impact on several business sectors. Partners Univ. Int. Innov. J. 1, 9–23. doi: 10.5281/zenodo.7644359

[B55] Goralski M. A. Tan T. K. (2020). Artificial intelligence and sustainable development. Int. J. Manag. Educ. 18:100330. doi: 10.1016/j.ijme.2019.100330

[B56] Gulati A. Saini H. Singh S. Kumar V. (2024). Enhancing learning potential: investigating marketing students behavioral intentions to adopt ChatGpt. Mark. Educ. Rev. 34, 201–234. doi: 10.1080/10528008.2023.2300139

[B57] Guo P. J. (2023). Six Opportunities for scientists and engineers to learn programming using AI Tools such as ChatGPT. Comput. Sci. Eng. 25, 73–78. doi: 10.1109/MCSE.2023.3308476

[B58] Gupta G. Singharia K. (2021). Consumption of OTT Media Streaming in COVID-19 lockdown: insights from PLS analysis. Vision 25, 36–46. doi: 10.1177/0972262921989118

[B59] Haberlin K. A. Atkin D. J. (2022). Mobile gaming and internet addiction: when is playing no longer just fun and games? Comput. Human Behav. 126:106989. doi: 10.1016/j.chb.2021.106989

[B60] Habibi A. Rasoolimanesh S. M. (2021). Experience and service quality on perceived value and behavioral intention: moderating effect of perceived risk and fee. J. Qual. Assur. Hosp. Tour. 22, 711–737. doi: 10.1080/1528008X.2020.1837050

[B61] Hair J. F. Ringle C. M. Sarstedt M. (2011). PLS-SEM: indeed a silver bullet. J. Mark. Theory Pract. 19, 139–152. doi: 10.2753/MTP1069-6679190202

[B62] Hair J. F. Risher J. J. Sarstedt M. Ringle C. M. (2019). When to use and how to report the results of PLS-SEM. Eur. Bus. Rev. 31, 2–24. doi: 10.1108/EBR-11-2018-0203

[B63] Halpern D. F. (2014). Critical thinking across the curriculum: A brief edition of thought & knowledge. Routledge. Available online at: https://www.taylorfrancis.com/books/mono/10.4324/9781315805719/critical-thinking-across-curriculum-diane-halpern (Accessed December 9, 2024).

[B64] Henseler J. Hubona G. Ray P. A. (2016). Using PLS path modeling in new technology research: updated guidelines. Ind. Manag. Data Syst. 116, 2–20. doi: 10.1108/IMDS-09-2015-0382

[B65] Hoffman D. L. Novak T. P. (1996). Marketing in hypermedia computer-mediated environments: conceptual foundations. J. Mark. 60, 50–68. doi: 10.1177/002224299606000304

[B66] Hong J.-W. Curran N. M. (2019). Artificial intelligence, artists, and art: attitudes toward artwork produced by humans vs. artificial intelligence. ACM Trans. Multimedia Comput. Commun. Appl. 15, 1–16. doi: 10.1145/3326337

[B67] Hsu C.-L. Lin J. C.-C. (2015). What drives purchase intention for paid mobile apps?–an expectation confirmation model with perceived value. Electron. Commer. Res. Appl. 14, 46–57. doi: 10.1016/j.elerap.2014.11.003

[B68] Huang Q. Zhang R. Lee H. Xu H. Pan Y. (2024). A study on customer behavior in online dating platforms: analyzing the impact of perceived value on enhancing customer loyalty. Behav. Sci. 14:973. doi: 10.3390/bs14100973 39457845 PMC11504637

[B69] Jo A. (2023). The promise and peril of generative AI. Nature 614, 214–216. doi: 10.1038/d41586-023-00340-6 36747115

[B70] Jo H. (2023). Understanding the key antecedents of users' continuance intention in the context of smart factory. Technol. Anal. Strateg. Manag. 35, 153–166. doi: 10.1080/09537325.2021.1970130

[B71] Karjaluoto H. Shaikh A. A. Saarijärvi H. Saraniemi S. (2019). How perceived value drives the use of mobile financial services apps. Int. J. Inf. Manage. 47, 252–261. doi: 10.1016/j.ijinfomgt.2018.08.014

[B72] Katz E. Blumler J. G. Gurevitch M. (1973a). Uses and gratifications research. Public Opin. Q. 37, 509–523. doi: 10.1086/268109

[B73] Katz E. Haas H. Gurevitch M. (1973b). On the use of the mass media for important things. Am. Sociol. Rev. 38, 164–181. doi: 10.2307/2094393

[B74] Kelton K. Fleischmann K. R. Wallace W. A. (2008). Trust in digital information. J. Am. Soc. Inf. Sci. 59, 363–374. doi: 10.1002/asi.20722

[B75] Kerr-Phillips B. Thomas A. (2009). Macro and micro challenges for talent retention in South Africa. SA J. Hum. Resour. Manag. 7, 1–10. doi: 10.4102/sajhrm.v7i1.157

[B76] Kim D. J. Ferrin D. L. Rao H. R. (2008). A trust-based consumer decision-making model in electronic commerce: the role of trust, perceived risk, and their antecedents. Decis. Support Syst. 44, 544–564. doi: 10.1016/j.dss.2007.07.001

[B77] Kim H.-W. Chan H. C. Gupta S. (2007). Value-based adoption of mobile internet: an empirical investigation. Decis. Support Syst. 43, 111–126. doi: 10.1016/j.dss.2005.05.009

[B78] Kim J. H. (2019). Multicollinearity and misleading statistical results. Korean J. Anesthesiol. 72, 558–569. doi: 10.4097/kja.19087 31304696 PMC6900425

[B79] Kim R. Song H.-D. (2022). Examining the influence of teaching presence and task-technology fit on continuance intention to use MOOCs. Asia-Pac. Educ. Res. 31, 395–408. doi: 10.1007/s40299-021-00581-x

[B80] Le T. M. D. Do H. T. N. Tran K. M. Dang V. T. Nguyen B. K. H. (2024). Integrating Tam and UGT to explore students' motivation for using ChatGPT for learning in Vietnam. Journal of Research in Innovative Teaching & Learning. Available online at: https://www.emerald.com/insight/content/doi/10.1108/JRIT-05-2024-0116/full/html (Accessed October 22, 2024).

[B81] Leung L. Wei R. (2000). More than just talk on the move: uses and gratifications of the cellular phone. Journal. Mass Commun. Q. 77, 308–320. doi: 10.1177/107769900007700206

[B82] Li Z. Liu Y. (2023). Analysis of the current situation of the research on the influencing factors of online learning behavior and suggestions for teaching improvement. Sustainability 15:2119. doi: 10.3390/su15032119

[B83] Liao Y.-K. Wu W.-Y. Le T. Q. Phung T. T. T. (2022). The integration of the technology acceptance model and value-based adoption model to study the adoption of e-learning: the moderating role of e-WOM. Sustainability 14:815. doi: 10.3390/su14020815

[B84] Lin K.-Y. Wang Y.-T. Huang T. K. (2020). Exploring the antecedents of mobile payment service usage: perspectives based on cost–benefit theory, perceived value, and social influences. Online Inf. Rev. 44, 299–318. doi: 10.1108/OIR-05-2018-0175

[B85] Liu C.-H. Horng J.-S. Chou S.-F. Yu T.-Y. Lee M.-T. Lapuz M. C. B. (2023). Digital capability, digital learning, and sustainable behaviour among university students in Taiwan: a comparison design of integrated mediation-moderation models. Int. J. Manag. Educ. 21:100835. doi: 10.1016/j.ijme.2023.100835

[B86] Liu X. (2020). Artistic reflection on artificial intelligence digital painting., in Journal of Physics: Conference Series, (IOP Publishing), 032125. Available online at: https://iopscience.iop.org/article/10.1088/1742-6596/1648/3/032125/meta (Accessed December 10, 2024).

[B87] Liu Y. X. Li H. Pan Y. (2025). Why do users trust Robotaxi? Analyzing user differences and adoption behavior from a socio-technical perspective. J. Retail. Consum. Serv. 87:104393. doi: 10.1016/j.jretconser.2025.104393

[B88] Maheshwari G. (2024). Factors influencing students' intention to adopt and use ChatGPT in higher education: a study in the Vietnamese context. Educ. Inf. Technol. 29, 12167–12195. doi: 10.1007/s10639-023-12333-z

[B89] Manyika J. (2017). Jobs Lost, Jobs Gained: Workforce Transitions in a Time of Automation. an Francisco, CA: McKinsey Global Institute.

[B90] Masrom M. Busalim A. Griffiths M. D. Asadi S. Mohd Ali R. (2024). The impact of excessive Instagram use on students' academic study: a two-stage SEM and artificial neural network approach. Interact. Learn. Envir. 32, 3546–3565. doi: 10.1080/10494820.2023.2184393

[B91] McQuail D. (1972). *The Television Audience: A Revised Perspective*. Sociology of Mass Communication/Longman. Harmondsworth: Penguin Books.

[B92] Mi L. Xu T. Sun Y. Zhao J. Lv T. Gan X. . (2021). Playing ant forest to promote online green behavior: a new perspective on uses and gratifications. J. Environ. Manage. 278:111544. doi: 10.1016/j.jenvman.2020.111544 33129025

[B93] Mollick E. R. Mollick L. (2022). New Modes of Learning Enabled by Ai Chatbots: Three Methods and Assignments. Available at SSRN 4300783. Available online at: https://papers.ssrn.com/sol3/papers.cfm?abstract_id=4300783 (Accessed December 4, 2024).

[B94] Moussawi S. Koufaris M. Benbunan-Fich R. (2023). The role of user perceptions of intelligence, anthropomorphism, and self-extension on continuance of use of personal intelligent agents. Eur. J. Inf. Syst. 32, 601–622. doi: 10.1080/0960085X.2021.2018365

[B95] Muir B. M. (1987). Trust between humans and machines, and the design of decision aids. Int. J. Man Mach. Stud. 27, 527–539. doi: 10.1016/S0020-7373(87)80013-5

[B96] Mulaik S. A. James L. R. Van Alstine J. Bennett N. Lind S. Stilwell C. D. (1989). Evaluation of goodness-of-fit indices for structural equation models. Psychol. Bull. 105:430. doi: 10.1037/0033-2909.105.3.430

[B97] Nagel J. K. Pidaparti R. M. (2016). Sig*nificance, prevalence and implications for bio-inspired design courses in the undergraduate engineering curriculum., in International Design Engineering Technical Conferences and Computers and Information in Engineering Conference, (American Society of Mechanical Engineers), V003T04A009*. Available online at: https://asmedigitalcollection.asme.org/IDETC-CIE/proceedings-abstract/IDETC-CIE2016/V003T04A009/257023 (Accessed December 9, 2024).

[B98] Nguyen T. Nguyen D. M. (2024). What will make Generation Y and Generation Z to continue to use online food delivery services: a uses and gratifications theory perspective. J. Hosp. Mark. Manag. 33, 415–442. doi: 10.1080/19368623.2023.2277731

[B99] Niu W. Zhang W. Zhang C. Chen X. (2024). The role of artificial intelligence autonomy in higher education: a uses and gratification perspective. Sustainability 16:1276. doi: 10.3390/su16031276

[B100] Overtoom C. (2000). Employability Skills: An Update. ERIC Digest No. 220. Available online at: https://eric.ed.gov/?id=ED445236 (Accessed December 4, 2024).

[B101] Palmgreen P. Wenner L. A. Rayburn J. D. (1980). Relations between gratifications sought and obtained: a study of television news. Communic. Res. 7, 161–192. doi: 10.1177/009365028000700202

[B102] Pan Z. Xie Z. Liu T. Xia T. (2024). Exploring the key factors influencing college students' willingness to Use AI coding assistant tools: an expanded technology acceptance model. Systems 12:176. doi: 10.3390/systems12050176

[B103] Peres R. Schreier M. Schweidel D. Sorescu A. (2023). On ChatGPT and beyond: how generative artificial intelligence may affect research, teaching, and practice. Int. J. Res. Mark. 40, 269–275. doi: 10.1016/j.ijresmar.2023.03.001

[B104] Ponte E. B. Carvajal-Trujillo E. Escobar-Rodríguez T. (2015). Influence of trust and perceived value on the intention to purchase travel online: integrating the effects of assurance on trust antecedents. Tour. Manag. 47, 286–302. doi: 10.1016/j.tourman.2014.10.009

[B105] Prasad Agrawal K. (2024). Towards adoption of generative ai in organizational settings. J. Comput. Inf. Syst. 64, 636–651. doi: 10.1080/08874417.2023.2240744

[B106] Qadir J. (2023). “Engineering education in the era of ChatGPT: Promise and pitfalls of generative AI for education,” in 2023 IEEE Global Engineering Education Conference (EDUCON) (Kuwait), 1–9. doi: 10.1109/EDUCON54358.2023.10125121

[B107] Ren X. Tong L. Zeng J. Zhang C. (2023). AIGC scenario analysis and research on technology roadmap of Internet industry application. China Commun. 20, 292–304. doi: 10.23919/JCC.fa.2023-0359.202310

[B108] Rousseau D. M. Sitkin S. B. Burt R. S. Camerer C. (1998). Not so different after all: a cross-discipline view of trust. AMR 23, 393–404. doi: 10.5465/amr.1998.926617

[B109] Rudolph J. Tan S. Tan S. (2023). ChatGPT: bullshit spewer or the end of traditional assessments in higher education? J. Appl. Learn. Teach. 6, 342–363. doi: 10.37074/jalt.2023.6.1.9

[B110] Ruggiero T. E. (2000). Uses and gratifications theory in the 21st century. Mass Commun. Soc. 3, 3–37. doi: 10.1207/S15327825MCS0301_02

[B111] Ryan R. M. Deci E. L. (2000). Self-determination theory and the facilitation of intrinsic motivation, social development, and wellbeing. Am. Psychol. 55:68. doi: 10.1037/0003-066X.55.1.68 11392867

[B112] Saeed Al-Maroof R. Alhumaid K. Salloum S. (2020). The continuous intention to use e-learning, from two different perspectives. Educ. Sci. 11:6. doi: 10.3390/educsci11010006

[B113] Shaikh Z. A. Khoja S. A. (2011). Role of ICT in shaping the future of pakistani higher education system. TOJET 10, 149–161.

[B114] Shukla S. (2021). M-learning adoption of management students': a case of India. Educ. Inf. Technol. 26, 279–310. doi: 10.1007/s10639-020-10271-8

[B115] Singh S. Singh N. Kalinić Z. Liébana-Cabanillas F. J. (2021). Assessing determinants influencing continued use of live streaming services: an extended perceived value theory of streaming addiction. Expert Syst. Appl. 168:114241. doi: 10.1016/j.eswa.2020.114241

[B116] Sobaih A. E. E. Elshaer I. A. Hasanein A. M. (2024). Examining students' acceptance and use of ChatGPT in Saudi Arabian higher education. Eur. J. Investig. Health Psychol. Educ. 14, 709–721. 10.3390/ejihpe14030047 PMC10969089 38534908

[B117] Song M. Xing X. Duan Y. Cohen J. Mou J. (2022). Will artificial intelligence replace human customer service? J. Retail. Consum. Serv. 66:102900. doi: 10.1016/j.jretconser.2021.102900

[B118] Stafford T. F. Stafford M. R. Schkade L. L. (2004). Determining uses and gratifications for the internet. Decis. Sci. 35, 259–288. doi: 10.1111/j.00117315.2004.02524.x

[B119] Sundar S. S. Kim J. (2019). “Machine Heuristic: When We Trust Computers More than Humans with Our Personal Information,” in proceedings of the 2019 chi conference on human factors in computing systems, (Glasgow Scotland: ACM), 1–9. doi: 10.1145/3290605.3300768

[B120] Taherdoost H. (2018). A review of technology acceptance and adoption models and theories. Procedia Manuf. 22, 960–967. doi: 10.1016/j.promfg.2018.03.137

[B121] Tlili A. Shehata B. Adarkwah M. A. Bozkurt A. Hickey D. T. Huang R. . (2023). What if the devil is my guardian angel: ChatGPT as a case study of using chatbots in education. Smart Learn. Environ. 10:15. doi: 10.1186/s40561-023-00237-x

[B122] Twum K. K. Ofori D. Keney G. Korang-Yeboah B. (2022). Using the UTAUT, personal innovativeness and perceived financial cost to examine student's intention to use E-learning. J. Sci. Technol. Policy Manag. 13, 713–737. doi: 10.1108/JSTPM-12-2020-0168

[B123] Ustun A. B. Karaoglan-Yilmaz F. G. Yilmaz R. Ceylan M. Uzun O. (2024). Development of UTAUT-based augmented reality acceptance scale: a validity and reliability study. Educ. Inf. Technol. 29, 11533–11554. doi: 10.1007/s10639-023-12321-3

[B124] Valentine A. (2013). “*Uses and gratifications of Facebook members 35 years and older,”* in The social media industries, (Routledge), 166–190. Available online at: https://api.taylorfrancis.com/content/chapters/edit/download?identifierName=doi&identifierValue=10.4324/9780203121054-10&type=chapterpdf (Accessed December 10, 2024).

[B125] Venkatesh V. Thong J. Y. L. Xu X. (2012). Consumer acceptance and use of information technology: extending the unified theory of acceptance and use of technology. MIS Q. 36, 157–178. doi: 10.2307/41410412

[B126] Wald R. Piotrowski J. T. Araujo T. van Oosten J. M. (2023). Virtual assistants in the family home. understanding parents' motivations to use virtual assistants with their child (dren). Comput. Hum. Behav. 139:107526. doi: 10.1016/j.chb.2022.107526

[B127] Wang S. Nah K. (2024). Exploring sustainable learning intentions of employees using online learning modules of office apps based on user experience factors: using the adapted UTAUT model. Appl. Sci. 14:4746. doi: 10.3390/app14114746

[B128] Wang S.-F. Chen C.-C. (2024). Exploring designer trust in artificial intelligence-generated content: TAM/TPB Model Study. Appl. Sci. 14:6902. doi: 10.3390/app14166902

[B129] Wang Y.-S. Yeh C.-H. Liao Y.-W. (2013). What drives purchase intention in the context of online content services? The moderating role of ethical self-efficacy for online piracy. Int. J. Inf. Manag. 33, 199–208. doi: 10.1016/j.ijinfomgt.2012.09.004

[B130] Wang Y.-Y. Wang Y.-S. (2022). Development and validation of an artificial intelligence anxiety scale: an initial application in predicting motivated learning behavior. Interact. Learn. Envir. 30, 619–634. doi: 10.1080/10494820.2019.1674887

[B131] Wei D. Chan L.-S. Du N. Hu X. Huang Y.-T. (2024). Gratification and its associations with problematic internet use: a systematic review and meta-analysis using use and gratification theory. Addict. Behav. 155:108044. doi: 10.1016/j.addbeh.2024.108044 38663155

[B132] Wu J.-H. Wang S.-C. Tsai H.-H. (2010). Falling in love with online games: the uses and gratifications perspective. Comput. Human Behav. 26, 1862–1871. doi: 10.1016/j.chb.2010.07.033

[B133] Wu M. Wang N. Yuen K. F. (2023). Can autonomy level and anthropomorphic characteristics affect public acceptance and trust towards shared autonomous vehicles? Technol. Forecast. Soc. Change 189:122384. doi: 10.1016/j.techfore.2023.122384

[B134] Wu R. Gao L. Lee H. Xu J. Pan Y. (2024). A study of the key factors influencing young users' continued use of the digital twin-enhanced metaverse museum. Electronics 13:2303. doi: 10.3390/electronics13122303

[B135] Xie C. Wang Y. Cheng Y. (2024). Does Artificial Intelligence Satisfy You? A meta-analysis of user gratification and user satisfaction with AI-powered chatbots. Int. J. Hum.-Comput. Interact. 40, 613–623. doi: 10.1080/10447318.2022.2121458

[B136] Xie J. Ye L. Huang W. Ye M. (2021). Understanding FinTech platform adoption: impacts of perceived value and perceived risk. J. Theor. Appl. Electron. Commer. Res. 16, 1893–1911. doi: 10.3390/jtaer16050106

[B137] Xiong Y. Shi Y. Pu Q. Liu N. (2024). More trust or more risk? User acceptance of artificial intelligence virtual assistant. Hum. Factors Ergon. Manuf. Serv. 34, 190–205. doi: 10.1002/hfm.21020

[B138] Yousafzai S. Y. Pallister J. G. Foxall G. R. (2003). A proposed model of e-trust for electronic banking. Technovation 23, 847–860. doi: 10.1016/S0166-4972(03)00130-5

[B139] Zhang C. Lu Y. (2021). Study on artificial intelligence: the state of the art and future prospects. J. Ind. Inf. Integr. 23:100224. doi: 10.1016/j.jii.2021.100224

[B140] Zhong J. Chen T. (2023). Antecedents of mobile payment loyalty: an extended perspective of perceived value and information system success model. J. Retail. Consum. Serv. 72:103267. doi: 10.1016/j.jretconser.2023.103267

