# Supplementary material for: Psychological drivers and barriers affecting students' sustained usage intention of AIGC tools in educational settings: an integrated perspective of Uses and Gratifications Theory and Perceived Value Theory
Source: Front Psychol. 2026 Aug 21;17:1892349. doi: 10.3389/fpsyg.2026.1892349 (PMC13541567; doi:10.3389/fpsyg.2026.1892349)
Supplement: Supplementary file 1 [file Supplementary_file_1.docx]

**Supplementary Figure S1.** Moderating effect of perceived trust on the relationship between information search and sustained usage intention.

**Supplementary Figure S2.** Moderating effect of perceived trust on the relationship between learning needs and sustained usage intention.

**Supplementary Figure S3.** Moderating effect of perceived trust on the relationship between perceived risk and sustained usage intention.

**Supplementary Figure S4.** Moderating effect of perceived trust on the relationship between perceived cost and sustained usage intention.

**Supplementary Table S1.** Assessment of Measurement Invariance.

| **Group** | **Con.** | **Conf. Inv.** | **Comp. Inv.** | | **PMI Est.** | **Equal Mean** | | **FMI Est.** | **Equal Var** | |
| --- | --- | --- | --- | --- | --- | --- | --- | --- | --- | --- |
|  |  |  | **C = 1** | **CI.** |  | **Diff.** | **CI.** |  | **Diff.** | **CI.** |
| Gender | CON | Yes | 1 | [0.998;  0.999] | Yes | 0.126 | [-0.168;  0.162] | No | -0.144 | [-0.149;  0.138] |
|  | EMP | Yes | 0.999 | [0.998;  0.999] | Yes | -0.124 | [-0.168;  0.165] | Yes | 0.217 | [-0.28;  0.266] |
|  | ENJ | Yes | 0.994 | [0.974;  0.991] | Yes | -0.13 | [-0.172;  0.177] | Yes | 0.27 | [-0.286;  0.282] |
|  | INI | Yes | 0.998 | [0.998;  0.999] | Yes | 0.076 | [-0.173;  0.163] | Yes | -0.165 | [-0.242;  0.229] |
|  | INS | Yes | 0.999 | [0.999;  1] | Yes | -0.114 | [-0.164;  0.166] | Yes | 0.157 | [-0.193;  0.168] |
|  | LN | Yes | 1 | [0.999;  1] | Yes | 0.071 | [-0.158;  0.175] | Yes | -0.114 | [-0.263;  0.225] |
|  | NOV | Yes | 1 | [0.998;  0.999] | No | 0.172 | [-0.172;  0.155] | No | -0.268 | [-0.221;  0.237] |
|  | PC | Yes | 0.98 | [0.609;  0.922] | Yes | -0.095 | [-0.171;  0.168] | Yes | 0.168 | [-0.234;  0.229] |
|  | PR | Yes | 0.988 | [0.94;  0.975] | Yes | 0.083 | [-0.175;  0.177] | Yes | -0.037 | [-0.217;  0.199] |
|  | PT | Yes | 0.999 | [0.996;  0.998] | Yes | -0.106 | [-0.179;  0.171] | Yes | 0.098 | [-0.212;  0.198] |
|  | PU | Yes | 1 | [0.998;  0.999] | Yes | -0.006 | [-0.183;  0.175] | Yes | -0.162 | [-0.341;  0.337] |
|  | SI | Yes | 0.999 | [0.998;  0.999] | Yes | -0.108 | [-0.159;  0.172] | Yes | 0.14 | [-0.266;  0.246] |
|  | SOA | Yes | 0.999 | [0.999;  1] | Yes | 0.125 | [-0.172;  0.171] | Yes | -0.201 | [-0.243;  0.234] |
|  | SUI | Yes | 1 | [1;  1] | Yes | 0.018 | [-0.171;  0.171] | Yes | -0.03 | [-0.143;  0.135] |
| Age | CON | Yes | 0.999 | [0.998;  0.999] | Yes | 0.037 | [-0.162;  0.166] | Yes | -0.075 | [-0.146;  0.153] |
|  | EMP | Yes | 0.999 | [0.998;  0.999] | Yes | 0.07 | [-0.169;  0.164] | Yes | -0.144 | [-0.26;  0.282] |
|  | ENJ | Yes | 0.997 | [0.972;  0.99] | Yes | -0.029 | [-0.167;  0.161] | Yes | 0.02 | [-0.27;  0.283] |
|  | INI | Yes | 0.999 | [0.998;  0.999] | Yes | 0.093 | [-0.167;  0.166] | Yes | -0.132 | [-0.229;  0.229] |
|  | INS | Yes | 1 | [0.999;  1] | Yes | -0.051 | [-0.16;  0.176] | Yes | 0.029 | [-0.176;  0.178] |
|  | LN | Yes | 1 | [0.999;  1] | Yes | 0.1 | [-0.174;  0.166] | Yes | -0.162 | [-0.24;  0.243] |
|  | NOV | Yes | 1 | [0.998;  0.999] | Yes | 0.084 | [-0.161;  0.17] | Yes | -0.084 | [-0.233;  0.23] |
|  | PC | Yes | 0.973 | [0.486;  0.915] | Yes | -0.011 | [-0.179;  0.156] | Yes | -0.027 | [-0.219;  0.262] |
|  | PR | Yes | 0.995 | [0.926;  0.972] | No | 0.201 | [-0.183;  0.168] | No | -0.231 | [-0.199;  0.22] |
|  | PT | Yes | 0.999 | [0.996;  0.998] | Yes | -0.105 | [-0.184;  0.183] | Yes | 0.078 | [-0.21;  0.219] |
|  | PU | Yes | 0.999 | [0.998;  0.999] | Yes | 0.025 | [-0.175;  0.179] | Yes | -0.09 | [-0.322;  0.373] |
|  | SI | Yes | 0.999 | [0.998;  0.999] | Yes | -0.046 | [-0.166;  0.159] | Yes | 0.118 | [-0.238;  0.264] |
|  | SOA | Yes | 0.999 | [0.999;  1] | Yes | 0.016 | [-0.167;  0.173] | Yes | -0.066 | [-0.246;  0.259] |
|  | SUI | Yes | 1 | [1;  1] | Yes | -0.002 | [-0.161;  0.172] | Yes | 0.031 | [-0.131;  0.141] |
| Education | CON | Yes | 1 | [0.998;  0.999] | Yes | 0.03 | [-0.167;  0.173] | Yes | -0.025 | [-0.163;  0.142] |
|  | EMP | Yes | 1 | [0.998;  0.999] | Yes | 0.032 | [-0.174;  0.184] | Yes | -0.053 | [-0.296;  0.28] |
|  | ENJ | Yes | 0.998 | [0.971;  0.99] | Yes | 0.117 | [-0.173;  0.176] | Yes | -0.221 | [-0.3;  0.257] |
|  | INI | Yes | 1 | [0.998;  0.999] | Yes | 0.123 | [-0.16;  0.179] | Yes | -0.194 | [-0.25;  0.228] |
|  | INS | Yes | 1 | [0.999;  1] | Yes | -0.137 | [-0.177;  0.175] | Yes | 0.126 | [-0.196;  0.18] |
|  | LN | Yes | 1 | [0.999;  1] | Yes | 0.071 | [-0.187;  0.171] | Yes | -0.095 | [-0.26;  0.24[ |
|  | NOV | Yes | 1 | [0.998;  0.999] | Yes | 0.075 | [-0.164;  0.175] | Yes | -0.082 | [-0.252;  0.221] |
|  | PC | Yes | 0.944 | [0.54;  0.916] | Yes | -0.03 | [-0.167;  0.179] | Yes | 0.034 | [-0.246;  0.228] |
|  | PR | Yes | 0.995 | [0.913;  0.971] | Yes | 0.044 | [-0.173;  0.182] | Yes | -0.061 | [-0.246;  0.206] |
|  | PT | Yes | 0.998 | [0.996;  0.998] | Yes | -0.074 | [-0.165;  0.18] | Yes | 0.05 | [-0.215;  0.184] |
|  | PU | Yes | 0.999 | [0.998;  0.999] | Yes | -0.117 | [-0.184;  0.172] | Yes | 0.181 | [-0.343;  0.33] |
|  | SI | Yes | 0.999 | [0.998;  0.999] | Yes | -0.025 | [-0.182;  0.168] | Yes | 0.041 | [-0.268;  0.26] |
|  | SOA | Yes | 1 | [0.999;  1] | Yes | 0.073 | [-0.162;  0.176] | Yes | -0.093 | [-0.276;  0.236] |
|  | SUI | Yes | 1 | [1;  1] | Yes | -0.027 | [-0.166;  0.179] | Yes | 0.008 | [-0.149;  0.136] |
| Experience with AIGC tools | CON | Yes | 0.999 | [0.998;  0.999] | Yes | 0.007 | [-0.172;  0.161] | Yes | 0.077 | [-0.148;  0.158] |
|  | EMP | Yes | 1 | [0.998;  0.999] | Yes | 0.022 | [-0.165;  0.178] | Yes | -0.069 | [-0.31;  0.249] |
|  | ENJ | Yes | 0.974 | [0.971;  0.989] | Yes | 0.019 | [-0.165;  0.171] | Yes | -0.049 | [-0.287;  0.263] |
|  | INI | Yes | 0.998 | [0.998;  0.999] | Yes | 0.017 | [-0.168;  0.183] | Yes | -0.015 | [-0.27;  0.212] |
|  | INS | Yes | 1 | [0.999;  1] | Yes | 0.018 | [-0.167;  0.169] | Yes | -0.089 | [-0.196;  0.182] |
|  | LN | Yes | 1 | [0.999;  1] | Yes | -0.011 | [-0.17;  0.183] | Yes | 0.025 | [-0.254;  0.228] |
|  | NOV | Yes | 0.999 | [0.998;  0.999] | Yes | 0.039 | [-0.183;  0.171] | Yes | -0.137 | [-0.249;  0.246] |
|  | PC | Yes | 0.987 | [0.653;  0.925] | Yes | 0.151 | [-0.17;  0.165] | Yes | No | [-0.243;  0.23] |
|  | PR | Yes | 0.985 | [0.908;  0.97] | Yes | -0.056 | [-0.165;  0.178] | Yes | 0.075 | [-0.231;  0.201] |
|  | PT | Yes | 0.997 | [0.996;  0.998] | Yes | -0.023 | [-0.167;  0.173] | Yes | -0.004 | [-0.213;  0.185] |
|  | PU | Yes | 0.999 | [0.998;  0.999] | Yes | 0.008 | [-0.171;  0.162] | Yes | 0.095 | [-0.316;  0.318] |
|  | SI | Yes | 0.999 | [0.998;  0.999] | Yes | -0.057 | [-0.163;  0.156] | Yes | 0.091 | [-0.244;  0.254] |
|  | SOA | Yes | 1 | [0.999;  1] | No | -0.193 | [-0.159;  0.173] | Yes | No | [-0.27;  0.221] |
|  | SUI | Yes | 1 | [1;  1] | Yes | -0.008 | [-0.17;  0.185] | Yes | 0.02 | [-0.154;  0.135] |
| Frequency of using AIGC tools | CON | Yes | 1 | [0.998;  0.999] | Yes | -0.015 | [-0.154;  0.156] | Yes | -0.019 | [-0.146;  0.145] |
|  | EMP | Yes | 0.999 | [0.998;  0.999] | Yes | 0.108 | [-0.171;  0.18] | Yes | -0.208 | [-0.284;  0.293] |
|  | ENJ | Yes | 0.998 | [0.972;  0.989] | Yes | -0.068 | [-0.171;  0.167] | Yes | 0.227 | [-0.268;  0.286] |
|  | INI | Yes | 0.999 | [0.998;  0.999] | Yes | 0.025 | [-0.171;  0.169] | Yes | -0.09 | [-0.223;  0.241] |
|  | INS | Yes | 0.999 | [0.999;  1] | Yes | 0.051 | [-0.167;  0.169] | Yes | -0.037 | [-0.182;  0.195] |
|  | LN | Yes | 1 | [0.999;  1] | Yes | -0.04 | [-0.169;  0.178] | Yes | 0.062 | [-0.242;  0.244] |
|  | NOV | Yes | 1 | [0.998;  0.999] | Yes | 0.123 | [-0.184;  0.181] | Yes | -0.208 | [-0.239;  0.253] |
|  | PC | Yes | 0.988 | [0.553;  0.917] | Yes | 0.094 | [-0.18;  0.18] | Yes | -0.137 | [-0.235;  0.258] |
|  | PR | Yes | 0.993 | [0.92;  0.975] | No | 0.234 | [-0.163;  0.171] | No | -0.232 | [-0.213;  0.214] |
|  | PT | Yes | 1 | [0.996;  0.998] | Yes | 0.009 | [-0.171;  0.173] | Yes | -0.009 | [-0.195;  0.2] |
|  | PU | Yes | 0.999 | [0.998;  0.999] | No | 0.166 | [-0.159;  0.171] | Yes | -0.325 | [-0.34;  0.32] |
|  | SI | Yes | 0.999 | [0.998;  0.999] | Yes | 0.05 | [-0.164;  0.172] | Yes | -0.086 | [-0.265;  0.255] |
|  | SOA | Yes | 1 | [0.999;  1] | Yes | 0.085 | [-0.173;  0.17] | Yes | -0.088 | [-0.227;  0.248] |
|  | SUI | Yes | 1 | [1;  1] | Yes | 0.096 | [-0.173;  0.174] | Yes | -0.084 | [-0.134;  0.142] |
